# 25 Years of Research in Human Lactation: From Discovery to Translation

**DOI:** 10.3390/nu13093071

**Published:** 2021-08-31

**Authors:** Donna Tracy Geddes, Zoya Gridneva, Sharon Lisa Perrella, Leon Robert Mitoulas, Jacqueline Coral Kent, Lisa Faye Stinson, Ching Tat Lai, Vanessa Sakalidis, Alecia-Jane Twigger, Peter Edwin Hartmann

**Affiliations:** 1 School of Molecular Sciences, The University of Western Australia, Crawley, WA 6009, Australia; zoya.gridneva@uwa.edu.au (Z.G.); sharon.perrella@uwa.edu.au (S.L.P.); leon.mitoulas@medela.com (L.R.M.); jacqueline.kent@uwa.edu.au (J.C.K.); lisa.stinston@uwa.edu.au (L.F.S.); ching-tat.lai@uwa.edu.au (C.T.L.); vanessasakalidis@gmail.com (V.S.); peter.hartmann@uwa.edu.au (P.E.H.); 2 Medela, AG, Lättichstrasse 4b, 6340 Baar, Switzerland; 3 Department of Pharmacology, University of Cambridge, Cambridge CB2 1PD, UK; ajt215@cam.ac.uk

**Keywords:** lactation, breastfeeding, human milk, milk composition, breast, infant feeding

## Abstract

Researchers have recently called for human lactation research to be conceptualized as a biological framework where maternal and infant factors impacting human milk, in terms of composition, volume and energy content are studied along with relationships to infant growth, development and health. This approach allows for the development of evidence-based interventions that are more likely to support breastfeeding and lactation in pursuit of global breastfeeding goals. Here we summarize the seminal findings of our research programme using a biological systems approach traversing breast anatomy, milk secretion, physiology of milk removal with respect to breastfeeding and expression, milk composition and infant intake, and infant gastric emptying, culminating in the exploration of relationships with infant growth, development of body composition, and health. This approach has allowed the translation of the findings with respect to education, and clinical practice. It also sets a foundation for improved study design for future investigations in human lactation.

## 1. Introduction

Breastfeeding is a major determinant of infant short- and long-term health, preventing acute infections and programming lower risk for chronic disease and is therefore considered to be a major public health focus [1]. Despite the wealth of information on the benefits to both the mother and the infant, global breastfeeding rates remain low, averaging only 41% [2]. To identify and address the issues faced by the breastfeeding dyad a foundational knowledge of the ‘norm’ is required. In the haste to provide support and solutions, an understanding of the basic science is frequently neglected in favour of the implementation of interventional trials that promise advances in care but are themselves based on an incomplete foundational understanding. The dearth of research into the fundamentals of human lactation over the latter part of the last century, combined with the lack of translation of the limited research actually performed into the medical model, has left clinicians little scope and tools to objectively assess breastfeeding problems. Evidence-based care, established from an understanding of the basic principles of lactation and mammary gland function is imperative to improve breastfeeding rates and subsequently the health and wellbeing of breastfeeding women, infants’, and their families.

**Figure 1:**
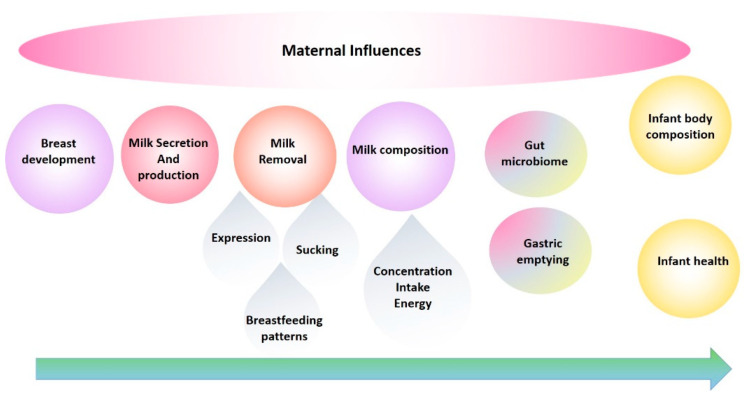
An overview of the structure of the review of our research programme from a biological systems perspective.

The evolution of our research program has been in direct response to the need to fill the translation gap and has been a journey from basic research into the anatomy, function of the breast and milk composition to multidimensional translational research elucidating mechanisms by which breastfeeding confers a multitude of benefits. These studies have provided the foundation for the development of successful interventions for those mothers experiencing lactation difficulties and have informed and reassured those women, particularly first-time mothers, that they are ‘normal’. This road has not been smooth, with findings that have disrupted the field requiring the reassessment of conventional dogma and change of deep-seated beliefs. Importantly, the rewards of change have been tangible and impactful for mothers and their babies. This review traces the main focal points of our research programme over the last 25 years and takes a biological systems approach by tracking breast milk from the mother (the breast) to the infant (Figure 1) [3] and aims to highlight and document these findings in the broader context of mammary gland function and the breastfeeding dyad, in order to provide a holistic platform of knowledge from which others may continue to work from in their pursuit to define better health outcomes for the next generation [4].

## 2. Breast Anatomy

**Figure 2:**
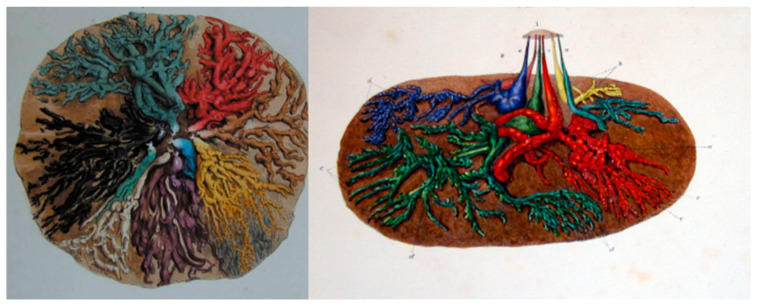
Sir Astley Cooper’s illustrations of the ductal system of the lactating breast. Duct were injected with coloured wax prior to dissection [6].

Despite the breast being the sole source of nutrition for the infant, this organ is rarely studied in its functional state. Interestingly, one of the most thorough investigations comes from over 180 years ago, by renowned anatomist and surgeon Sir Astley Cooper. This work, published in 1840, is a remarkable description of the anatomy of the lactating human breast and was performed in response to a request to document diseases of the breast. Cooper asserted that knowledge of basic anatomy was critical to the understanding of breast anomalies and disease. As such, he proceeded to meticulously investigate the anatomy of the breast, sourcing breasts for dissection from the corpses of women who had died during lactation. For over 150 years, Cooper’s work was the most complete examination of the lactating breast with his work most notable for its depictions of the ductal structures of the breast, obtained by the delicate dissection of the mammary ductal system after the injection of coloured wax into the ducts of the nipple [5] (Figure 2). His contribution is memorialised via the eponymously named Cooper’s ligaments that provide a framework for the tissues of the breast.

The macro-anatomy of the lactating breast may be described by its external and internal components. The external structures: skin, areola, Montgomery tubercles and nipple exhibit wide variation in size and colour amongst women. Internally the main tissues of the breast consist of the glandular-ductal system, adipose tissue and extracellular matrix supported by the fibrous Cooper’s ligaments. Like the external structures, the internal structures of the breast are also variable between women in terms of volume of tissues. At the cellular level, the glandular-ductal system consists of hundreds of alveoli, each comprised of a bilayer of luminal and basal cells. The luminal cells consist of mammary epithelial cells or lactocytes that serve to synthesise and secrete milk into the lumen of the alveolus. The alveoli are surrounded by a network of myoepithelial cells that contract at milk ejection (ME), to expel the milk from the alveoli into the milk ducts, thus making it available to the infant.

As originally depicted by Cooper, the ductal structures within the breast are arranged in a complex network. Each alveolus is connected to this network via a ductal outlet and the diameters of these lactiferous ducts progressively increase in size as they get closer to the nipple where they narrow once more as they pass through the nipple, therefore providing a mechanism to prevent the escape of milk. A key aspect of Cooper’s work was the presence of lactiferous sinuses, ducts distended with milk just beneath the areolae. According to Cooper, the areola was considered an extension of the nipple and had three main functions: to give a greater adhesion to the infant’s lips; add to the sensibility and connectivity of the areola with mammary gland; and an area embraced by the infant’s lips and into infant’s mouth from where the lactiferous sinuses behind the areola can be emptied by the pressure of the lips of the infant [5]. In other words, it was believed that milk transfer to the infant was executed by the lips of the infant when applying a compressive pressure around the areola. This observation highlighted Cooper’s assertion that knowledge of the anatomy of the mammary gland was important for understanding function and formed the basis of milk removal theory for over a century.

The contribution of advanced imaging techniques such as the non-invasive approach of ultrasonography built upon the remarkable work performed by Cooper and has, at the same time, updated some of the interpretations of the previous model, leading to our current understanding of breast anatomy [7] and has impacted the clinical appraisal of milk removal. Ramsay et al. used ultrasound to image both breasts to determine the number of main ducts, duct morphology and distribution of glandular and adipose tissue. Milk ducts, observed in a resting state, were identified at the base of the nipple and were superficial and easily compressible lending support to anecdotal reports of tight bras or seat belts blocking ducts and causing milk stasis [8]. The average number of ducts exiting the nipple was nine with a range from as few as four to as many as 18, less than the 20–25 typically stated and consistent with observed milk jets during pumping [9]. Resting milk duct diameters are small (2 mm, range 1.0–4.4 mm) and similar in size to non-lactating duct diameters except for the significant dilation observed at ME. The lower number and smaller diameters of the ducts compared to Cooper’s work suggests that for some women who exhibit poor lactation outcomes after surgical procedures, it is the severing of the majority of ducts that that is contributing to low supply issues. Importantly, neither the number of the ducts nor the mean diameter of the ducts were related to nipple diameter providing evidence that the internal breast morphology is not predicted by the external anatomy of the breast [7].

During pregnancy there is an increase in size, length and elasticity of the nipple which varies widely between women [10] with reported nipple diameters of 15.7 ± 1.8 mm and 15.8 ± 2.8 mm for the left and right nipples respectively [11]. As the infant’s mouth accommodates the nipple, the size and shape of the nipple are clinically important for the infant to attach adequately to the breast.

**Figure 3:**
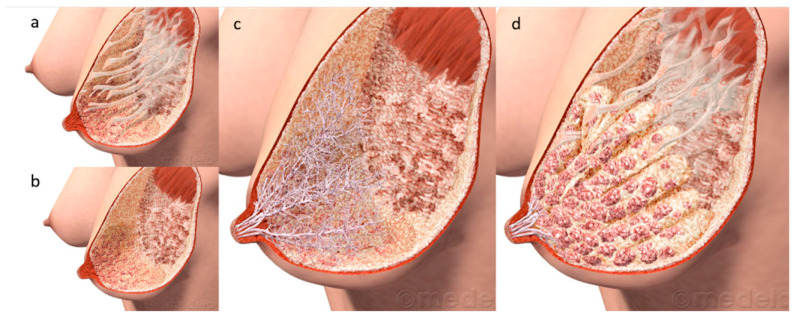
New understanding of the anatomy of the lactating breast. (**a**) Coopers ligaments (**b**) superficial and retromammary fat layers (**c**) milk duct system (**d**) macro anatomy of the breast. © Medela AG 2006. Used with permission.

Importantly, Ramsay’s study highlighted the absence of lactiferous sinuses superficial to the areolae, a result in contrast to Cooper’s observations but one that was subsequently confirmed by Gooding et al. with three-dimensional ultrasound imaging [12]. Furthermore and again in contrast to Cooper‘s reports, which have underpinned the long-held understanding of breast anatomy and breastfeeding physiology, all of the observed ducts branched from the areola, under the nipple and only very small volumes of milk (1–10 mL) could be expressed from them in the absence of ME, suggesting the function of the ducts is to transport rather than store milk. Cooper’s injection of coloured wax/glue through the nipple ductal outlets combined with our observation that the ducts are extremely elastic likely provided for an expanded wax representation of the ductal system leading to the erroneous depiction of lactiferous sinuses and an overestimation of the volume of milk that would be stored under the areola. These findings explain the variation in volumes of milk removed both in the colostral phase where the milk volume is low and viscous (Figure 3) and prior to milk ejection in established lactation.

As mentioned previously, internally the breast consists primarily of glandular and adipose tissue, with some connective tissue as well as the myoepithelial (smooth muscle) network surrounding the alveoli. The proportions of glandular and adipose tissue were found to be in an approximate ratio of 2:1 and was similar within women. However, there is wide variation between women for glandular tissue 65 ± 11% (45–83%) and 63 ± 9% (46–83%), and adipose tissue (37 ± 9% (16–51%) and 35 ± 12% (9–54%) for right and left breasts respectively). Interestingly, 24 h milk production was not correlated with the amount of glandular tissue, number of milk ducts, duct diameters or storage capacity of the breast. Given that storage capacity is a reflection of the amount of glandular tissue, the finding that some breasts are comprised of up to 50% adipose tissue suggests that it is possible that some larger breasts may actually have a lower storage capacity than otherwise expected and may account for the unexpected frequent feeding observed in these dyads. In total, these findings reaffirm the evidence that for physiologically normal breastfeeding women, milk production is driven by the interplay of the infant’s appetite, frequency and efficacy of milk removal, and maternal anatomy. This seminal work has redefined our understanding of breast anatomy in lactation, thus impacting our knowledge of breastfeeding physiology and management of breastfeeding problems.
**Breast Anatomy** Number of ductal openings on the nipple is 4–18 (previously 15–20) Milk ducts branch close to the nipple The conventionally described lactiferous sinuses do not exist Milk ducts can reside close to the skin surface and are easily compressible Most of the glandular tissue is found within 30 mm of the nipple

## 3. Milk Ejection

The ME reflex is highly conserved across all mammalian species from monotremes and marsupials to the placental mammals [13]. In women it is critical to the success of lactation as little to no milk can be removed from the breast prior to ME, thus milk synthesis is downregulated in the absence of ME.

The ME reflex is a neuroendocrine reflex is triggered by nipple stimulation sending neural impulses to the hypothalamus stimulating the release of oxytocin from the posterior pituitary gland into the maternal bloodstream. Oxytocin then travels via the maternal circulation to the mammary gland, causing the contraction of the myoepithelial cells surrounding the alveoli [14] forcing milk through the ductal system towards the nipple for removal by the infant or a breast pump [15]. The force of the milk flow dilates the ducts and increases positive pressure within the ducts [16,17,18,19]. This reflex, occurs within 60 s and 90 s for breastfeeding and pumping respectively [17,20] in both breasts simultaneously [21], with slight discrepancies due to binding of oxytocin to fuller alveoli [22]. Milk ejection is transient lasting 45 s to 3.5 min [17,23] with multiple MEs measured during a feed or pumping session (breastfeeding: 2.5; breast pumping: 4.4) [18,24].

**Figure 4:**
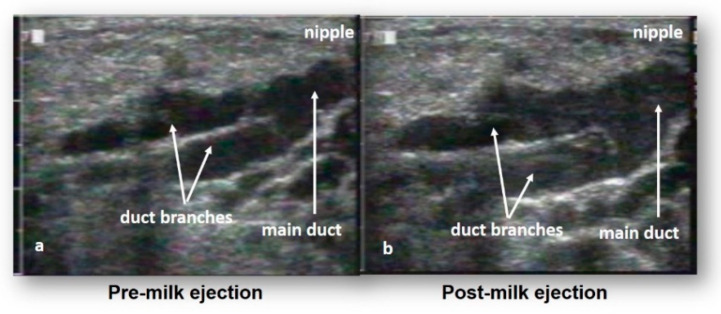
Ultrasound images of milk ducts pre-milk ejection (**a**) and post-milk ejection (**b**). Milk ducts are imaged as black (anechoic). Post milk ejection the ducts are expanded with breast milk (Geddes et al., unpublished).

Employing measurement of ME with ultrasound imaging (Figure 4) or measurement of milk flow rates [8,18,21] we have shown that while ME is individualised it is highly conserved within women [25] such that ME patterns remain consistent between breasts, between breastfeeding and pumping, with different pumping patterns, between weeks and across lactation (9 months) [26]. Thus, it appears that differences in the source of stimulation of the breast/nipple in women in established lactation does not alter the ME reflex. Replication of the ME patterns in subsequent lactations has also been observed, [27] building on the hypothesis that ME is innate. This finding also suggests that it is the requirement of the infant to adapt to the mother’s ME pattern and changing rates of milk flow in order to remove milk effectively and efficiently from the breast [28]. The volume of milk removed by the infant is likely then governed not only by infant appetite but also by the combination of the volume of milk stored in the breast and its availability as a result of the ME pattern, as an increasing number of MEs during a breastfeed is related to higher volume of milk consumed [15].

In relation to milk removal during pumping, the majority is removed during the first two MEs (76–86%) [29] emphasising that the switch from ‘stimulation’ mode to ‘expression’ mode should be made as soon as ME occurs to maximise the removal of milk early in the expression when intraductal pressure is at its highest [16]. Further, when the expression pattern was changed mid pumping, it did not always coincide with a ME suggesting the change of pumping stimulus does not necessarily increase the likelihood of precipitating a ME [30].

To date we have utilised ultrasound imaging and milk flow measurement [16,26,31] as well as having explored alternative technologies such as bioimpedance spectroscopy and thermal imaging [31,32] for the detection of ME. Bioimpedance applied to the pumped breast detected the initial ME 90% of the time [23]. Temperature however was not successful as we were unable to detect a change in nipple temperature on the non-pumped breast during breastfeeding or pumping, [33] despite the hypothesis that the release of oxytocin would result in a change of nipple temperature. It will be important to further pursue these detection technologies as development of methods to identify and assess ME may provide breastfeeding mothers and clinicians with biofeedback on ME function as well as supporting the development of breast milk expression innovations.
**Milk ejection** Milk ejection is critical for milk removal Milk ejection patterns are unique to the individual Milk ejection patterns do not change with stimulus (breastfeeding or pumping or different pumping patterns) Milk ejection patterns do not change over lactation Milk ejection patterns do not change between lactations

## 4. Secretory Activation

Secretory activation (SA) marks the beginning of copious milk production and occurs within 48–72 h after birth commensurate with the rapid decline in progesterone after the delivery of the placenta [34]. The sensation of breast fullness lags the biochemical changes associated with SA by up to 30 h [35]. The most marked changes in milk composition at SA are the decreases in milk sodium and protein concentrations accompanied by the increases in lactose and citrate concentrations [34]. These changes are due to both closure of tight junctions between lactocytes (sodium) as well as increased milk synthesis (lactose, protein, citrate) [35]. Delayed SA, i.e., beyond 72 h, has been associated with suboptimal lactation outcomes such as lower milk production and reduced duration of breastfeeding. While long labours, caesarean section, and obesity have been associated with delayed SA [36,37] we found no evidence of delayed SA in women delivering by vaginal or caesarean section nor with analgesic administration during caesarean births [38]. Similarly, Cregan et al. found no difference in concentration of milk citrate, lactose, protein, and sodium from mothers of term and preterm infants [39]. However, there was greater variation in these markers for preterm mothers and milk production was related to how many markers were in the normal range by Day 5 [39] in that women with fewer markers in the normal range had the lowest milk productions. The significance of these data are that previously SA was considered a discrete event with failed SA attributed to either hormonal causes, e.g., no drop in progesterone due to retained placenta, or glandular causes, e.g., insufficient mammary tissue [40]. The observation by Cregan et al., that mothers can have some markers in the normal range and others not, indicated for the first time that the concept of a compromised SA was possible. Hoban et al. have extended this work to daily sampling for 14 days post-partum for mothers delivering preterm and found that 40% of the samples had normal concentrations of SA biomarkers with only a third of women reaching adequate milk volume by day 14 [41,42]. Interestingly, sodium appeared to most predictive of low milk volumes [42]. In this connection, small ion-selective probes are readily available to measure sodium in milk and have been validated against bench biochemical methods providing the potential for a point-of-care instrument [43] to monitor the initiation of lactation. This ability to measure markers early in lactation provides the clinician an opportunity to increase support and care of those mothers with known risk factors to maximise their likelihood of successfully establishing a milk production.

Early initiation of breastfeeding and/or pumping has been shown to reduce delayed SA in at risk mothers [44]. In addition, for women pumping their milk in the first 3 days after term and preterm birth, the application of an initiation pattern modelled on the sucking characteristics of a term infant has been shown to improve milk production [45,46]. These findings all underscore the importance of frequent and efficient milk removal (feeding or pumping when separated from the infant) in the first days after birth to establish a robust milk production to meet the needs of the infant [42,46,47].
**Secretory activation is marked by**Closure of tight junctions between the lactocytesBiochemical changes in milk at 48–72 hSensations of fullness of the breast (later than biochemical changes)**Secretory activation**Is often delayed in at risk mothersIs facilitated with frequent and efficient milk removal in the early post-partum which increases milk production

## 5. Milk Production

Whilst the growth, health and development of the breastfed infant depends upon an adequate milk production it is rarely measured in both the research and clinical setting despite clinician estimates of milk transfer being shown to be inaccurate [48] and perceived low milk supply being a major cause of early weaning [47]. Indeed, we have found that perceived insufficient milk supply accounts for close to half (44%) of the women attending a tertiary hospital breastfeeding centre for advice. For these mothers, the major concern was that the infant did not seem satisfied after a breastfeed. This perception of an insufficient milk supply improved in a small subset of mothers who were surveyed after receiving targeted lactation advice despite lack of confirmation of increased milk production [49]. Three methods are available to measure milk production: deuterium dilution [50,51], test weighing [52] and hourly pumping [53,54], with all methods having advantages and disadvantages. Deuterium dilution does not provide information about breastfeeding patterns and has a long analysis time and requires specialist equipment, precluding its use as a routine clinical tool. However, test weighing allows investigation of breastfeeding patterns as well as the effectiveness of milk removal by the infant with instantaneous results. The hourly pumping technique offers an alternative in cases where a mother is not able to carry out test weighing but requires the infant to be fed expressed milk [53,55] and still needs more formal validation before it can be considered for routine use [54]. As such, 24 h test weighing has underpinned many of the research studies in our laboratory.

**Figure 5:**
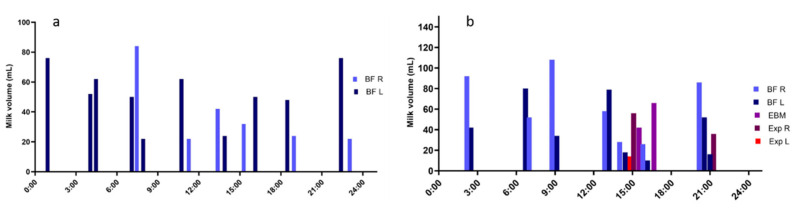
(**a**) Milk production of 792 mL with infant intake consisting of paired and single breastfeeds. (**b**) Milk production of 967 mL where the infant consumed 849 mL from the breast, 88 mL was expressed (Exp R + Exp L) and the infant consumed 108 mL of expressed breast milk (EBM).

After birth frequent and effective milk removal is critical to the establishment and maintenance of milk production [56,57], with milk production established by week 1 to 2 [58] and production at week 2 being indicative of production at 6 weeks [59]. In a landmark paper by Kent et al. normal breastfeeding patterns and total fat intake were documented for mothers exclusively breastfeeding infantsbetween one and six months of lactation and showed a wide variation of milk productions while still providing adequate milk and fat intakes (Table 1, Figure 5) [60]. The results suggest that for this population of mother, advice should not be given to schedule breastfeeding or to shorten or prolong feeds and that prioritizing so call ‘hind milk’ over ‘fore milk’ is unfounded. Interestingly, it was shown that night feedings (10 p.m.–4 a.m.) were common, with 100% of infants under 9 weeks of age feeding at night. Furthermore, those infants consumed a significant portion of their 24 h intake (20%) during this night feed timeframe, highlighting caution when considering ‘sleep training’ as this could impact not only milk intake but also down regulate milk production. This work was extended to show that milk production did not change significantly over the first 6 months of lactation in exclusively breastfeeding women [61] indicating that recommendations to increase milk intake and energy intake according to the infant’s age is not appropriate for fully breastfed infants. Intriguingly, several studies have shown one breast is often more productive than the other with that breast often the right breast [60,62,63,64] whilst others have found no difference between the breasts [65]. Given these differences in milk production/milk intake from each breast, it is important that the calculation of infant intake of any milk component should be made for each breast separately [63,65]. 

Whilst these data documented the ‘norm’, the 24 h test weighing method was used at a tertiary hospital breastfeeding centre for mothers with breastfeeding problems to determine the proportion of mothers who actually had low milk production (defined as less than 440 mL/day). It was found that 46% of mothers had low production between days 6 and 13 and 45% of mothers had a low milk production between days 14–28 respectively [58]. When viewed in light of the previously mentioned 44% of mothers with a perception of low milk supply, it shows the importance of being able to accurately assess milk production in these women as often their perceptions can be well founded. However, test weighing is often met with resistance because of the notion that maternal confidence will be negatively impacted. The results of a survey of 203 women dispelled this idea, in that 66% of women were still confident and 11% improved their confidence in breastfeeding after completing a 24 h production measurement. Importantly, those that lost confidence all had milk productions well below 478 mL/24 h (minimum documented milk production in Kent et al., 2006 [60]) suggesting that this method can be used routinely by clinicians to instigate clinical intervention when necessary [66] and without fear of negatively impacting maternal confidence. This is aptly demonstrated in a case-study of a breastfeeding baby with Down’s Syndrome where due to initial concerns in hospital the infant was supplemented with formula and expressed milk in but returned home exclusively breastfeeding. Maternal concerns about milk production were confirmed with 546 mL/24 h at 4 weeks and this improved with additional expressions to 819 mL/24 h at 10 weeks. The increase in milk intake was reflected in adherences to the appropriate growth trajectory. By 24 weeks this mother was fully breastfeeding 924 mL/24 h [67]. Further, we have found that women who have perceived insufficient milk supply and received clinical lactation support generally had improvement in their perceptions. However, infant unsettledness after a feed and formula supplementation was still prevalent indicating that measures of milk production may be useful in determining patterns of milk removal [49].

Many factors are believed to impact milk production such as age, parity, and infant sex. Recent studies have shown no reduction of milk production with increasing maternal age with those between 15 and 37 years and [68] those between 23 and 42 years having an average milk production of 750 to 800 mL [69]. Similarly, there is no strong evidence yet of an increased milk production in women with higher parity, despite the demonstration in an animal model of epigenetic memory in mammary epithelial cells in subsequent lactations resulting in a more robust onset of lactation and volume [70]. It is recognised however, that infant sex appears to influence milk production in that boys on average consume 80–100 mL more than girls and this is also reflected in the storage capacity of the breast being greater for mothers of boys [60].

## 6. Breast Anatomy and Milk Production

Breast hypoplasia (unilateral or bilateral) [71] impacts up to 25% or women [72] and typically results in under or disorganised breast development resulting in limited milk supply despite efforts to increase frequency of feeding and expression. Whilst hypoplasia is linked to genetic causes, such as zinc transporter mutations [72] there are currently no tests, genetic or otherwise to confirm hypoplasia and its link to low milk production.

The storage capacity of the breast is highly variable between women ranging from 74 to 382 mL (mean around 180 mL) [60]. The more milk available to the infant in the breast the greater the volume the infant tends to receive thus women with smaller storage capacities may need to feed more frequently than those with higher storage capacities. In cases of very high frequency of feeding measurement of 24-h milk production would be warranted.

Low production is also documented with women that have had breast surgery, trauma, or nipple piercing. These procedures often sever milk ducts, thus disrupting milk removal and, over time, potentially isolating glandular tissue, causing milk stasis and ultimately apoptosis and regression of the tissue. Alternatively, in some instances breast implants may have been inserted into breasts that were already hypoplastic [73,74,75,76].

## 7. Breast Physiology

Typically, breast growth is obvious in pregnancy due to proliferation of glandular tissue and the differentiation lactocytes to produce milk [10,77]. Breast size however is not indicative of milk production although women with smaller breasts are more likely to have smaller storage capacities and therefore feed more frequently than those women with larger breasts [77].

Increasing evidence also suggests that pregnancy complications such as gestational diabetes mellitus, preeclampsia, gestational hypertension [78], and fetal growth restriction [79] are associated with shorter durations of breastfeeding. While caesarean section has been associated with delayed initiation of breastfeeding, in those women that breastfeed secretory activation [38] and any breastfeeding at 6 months appears to not differ between caesarean section and vaginal delivery [80]. In addition, women experiencing postpartum haemorrhage, including those that receive a blood transfusion in hospital, also show reduced breastfeeding at discharge, irrespective of haemoglobin concentration pre-transfusion and persistence of anaemia post transfusion [81,82].

Mastitis or inflammatory lactating breast conditions often result in increased permeability of the alveolus, evidenced by increased human milk (HM) sodium, chloride, lactoferrin, serum albumin concentrations and decreased HM lactose and glucose as well as increased 24-h excretion of lactose, blood, and milk C-reactive protein [83,84,85]. Furthermore, we have observed greater numbers of immune cells and expression of immune proteins such as granzyme B [86] in the milk of women with mastitis. Many of the changes in milk composition seen with mastitis are observed with perceived and measured low milk supply supporting maternal reports of reduced milk supply with mastitic episodes [83].

Blocked ducts are also commonly experienced during lactation and can be associated with engorgement and inflammatory symptoms [87], yet little is understood about the causes and effective treatments [88]. Whilst a blocked duct may resolve in 24 h with increased milk removal and massage, non-resolution of these breast masses should cause concern and initiate imaging investigations such as ultrasound to exclude other causes such as fibroadenomas, cysts, lymph nodes and malignancy [89].

## 8. Medications

Few medications have been shown to impact milk production either positively or negatively Pseudoephedrine hydrochloride a common over-the-counter decongestant medication, was found to reduce milk production by up to 24%, due to a potential reduction in maternal serum prolactin levels [90]. Conversely a pharmaceutical galactagogue such as domperidone, which increases prolactin, can provide a modest increase in milk production of on average 100 mL, in a dose dependent manner [91]. Although it is not clear whether this increase in milk volume is confounded by the increased emptying of the breast [92]. Indeed large increases in milk production by domperidone, may not be achievable during established lactation, due to the absence of a relationship between prolactin and short-term rates of milk synthesis [64]

## 9. Factors Impacting Milk Removal

Frequent, effective milk removal from the breast is the mainstay for the establishment and maintenance of milk production. For the breastfeeding dyad a feeding frequency of at least 7 × 24 h in the first 2 weeks after birth is associated with establishment of an adequate milk production [57,93] while in established lactation breastfeeding frequency is 11 breastfeeds or 8 breastfeeding sessions [60]. While ‘normal’ feeding frequency has been characterised there are few methods by which to gauge efficacy of milk removal. The volume of milk removed from the breast is not indicative of effectiveness of emptying because it does not consider the amount of milk stored in the breast, or ‘degree of breast fullness’ and this varies significantly over a 24 h period within and between women. A more accurate estimation is the percentage of available milk removed (PAMR) from the breast which; is estimated from the breast storage capacity and degree of fullness of the breast before the feeding/expression [94]. On average the term infant removes 67% of the available milk during a breastfeed whereas expression with a hospital grade electric breast pump removes on average 55–75% [95,96,97,98]. Data for the effectiveness of personal use pumps and hand expression are not available, although evidence suggests that significantly higher milk volumes are expressed with a hospital grade pump when compared to hand expression [99].

Many factors potentially impact milk production (Table 2) for example mother and infant separation in hospital restricts access to the breast for the infant to breastfeed, decreases skin-to-skin contact and reduces breastfeeding [100]. Indeed, in the recent COVID pandemic women with COVID-19 were separated from their infants initially until it was realised that vertical transmission of the virus through milk did not occur [101]. In cases of separation, it is critical that the mother continues to effectively remove milk from the breast either by hand expression or by using a pump to establish milk production as reduced feeding in the first 2 weeks has also been associated with lower milk production [59]. The mothers of preterm infants often experience a quadrupled burden with a pregnancy complication (e.g., pre-eclampsia), disrupted development of the breast, separation from their infant, and inability of the infant to remove large volumes of milk from the breast thus rely expression of milk to establish and maintain their milk supply. For exclusively pumping women, so often the situation for mothers of preterm infants, there is limited information on the frequency of milk removal required to maintain an adequate production. Our study of exclusively pumping preterm mothers indicates that extension of pumping intervals slows the rate of milk synthesis such that the synthetic rate was significantly slower beyond 7 h. In this connection, the amount of milk removed at each pumping episode increased from 2 to 6 h intervals, reaching a plateau at 7 h. Further, while it appears that a minimum of 5 expressions/24 h is necessary to maintain milk production, increased frequency is warranted for those that have not established a full production given the high observed inter-individual variation [102].

The frequency and effectiveness of feeding at the breast can also be hampered by several intra-oral anomalies. Our studies have shown that some infants with ankyloglossia (tongue tie) are able to sustain an adequate milk production when breastfeeding [103] whereas, others are unable to [104,105]. In this regard, frenotomy for an anterior tongue tie has been shown to improve production, albeit in a small number of babies [104]. More recently cases of tongue tie have appeared with increased frequency, most likely driven by the increased diagnosis of posterior tongue tie. However, the subsequent increase in frenotomy [106] have raised concerns about increased post-surgical complications [107]. Furthermore, preliminary evidence suggests that milk production can remain compromised in a number of infants after frenotomy for posterior tongue tie, indicating other potential causes of insufficient supply [108]. These studies have provided the impetus for revision of the anatomy of the lingual frenulum [109,110,111], definitions and clinical protocols for ankyloglossia [112], and attempts with non-surgical methods to improve breastfeeding in infants diagnosed with ankyloglossia [113].

There are other infant anomalies that typically affect the infant’s ability to generate sufficient vacuum, which we have shown to be instrumental for effective milk removal [114]. Typically, infants that have difficulty creating vacuum such as those born preterm, infants with low tone, Down’s syndrome, and cleft lip/plate have reduced milk removal [69,115,116]. In contrast, those infants of mothers experiencing pain during breastfeeding often apply very strong vacuums (Baseline vacuum: control: −52 mmHg, Pain: −95 mmHg; Peak vacuum: control: −171 mmHg; Pain: −238 mmHg) during breastfeeding and milk volume is reduced (Control: 78 mL; Pain: 55 mL) either due to altered tongue dynamics [117], the strong vacuum itself or reduction of the efficacy of milk removal due to diminished oxytocin release at ME reflex as a result of the pain [118].

Pump settings influence effectiveness and efficiency of milk removal. For example, it is only possible to remove substantial volumes of milk from the breast during ME. Thus, the effective simulation of ME has been a focus of both manual and electric pumping where patterns have been designed to mimic the breastfeeding infant’s rapid sucking at the beginning of the feed [119]. We have tested multiple patterns and found that faster patterns (>100 cycles/min) elicited ME more quickly than slower patterns [20]. Interestingly there did not seem to be an effect of stronger vacuum in the time to trigger an ME. However, this was not assessed systematically within a mother, thus it would be prudent for each mother to set the pump at her maximum comfortable vacuum.

The strength of both infant intra-oral vacuum [95,114] and pump expression vacuum are implicit in efficacious milk removal [29]. Stronger vacuums during pumping increase milk flow rate, volume pumped and the cream content at the end of the expression session as well indicating better breast emptying. Thus it is important for women that need to pump their milk to test their maximum comfortable vacuum over time as often women are more sensitive in the immediate days post-partum [120]. Efficacy of milk removal has been shown to be significantly higher with breast pumps compared to hand expression [115] most likely due to the absence of vacuum during hand expression and the reliance on internal positive pressure within the milk ducts only [16]. In this connection, weak vacuums applied by the infant during breastfeeding may also negatively impact milk removal, requiring additional expression to achieve adequate breast emptying to maintain or establish milk production [67,116]. Furthermore, double pumping is more efficacious when compared to sequential single pumping, with milk removed during more milk ejections during double pumping [21].

For the mother using a breast pump, the breast shield is the interface between the pump and the breast. As such, shield shape, size and characteristics can influence effectiveness of milk removal [19,121]. Based on the fact that warmth is applied to the nipple by the breastfeeding infant, we investigated whether the application of warmth to the breast shield would promote more efficient milk removal [95]. We found that the time to remove 80% of the milk pumped was decreased (4.8 versus 6.8 min) indicating many women could pump for shorter periods of 5–8 min under these conditions. Ultrasound imaging supported the results by showing a significant reduction in nipple duct diameter with cold versus warm ultrasound gel.

In more recent pumping and breastfeeding studies, we measured nipple haemodynamics using infrared imaging as a proxy for blood flow [33,122]. Nipple temperature increased during both breastfeeding and pumping. For each 1 °C increase in nipple temperature, an additional 10 mL of milk was removed during pumping. It is not clear whether this increase in temperature is due to the vasodilatory action of oxytocin, local vasodilators, relaxation of the muscle fibres in the nipple or heating caused by nipple movement in the breast shield during pumping [98,123,124]. Further, infrared imaging may have potential to identify atypical nipple responses to feeding/pumping, nipple oedema, and inflammation that may hinder effective milk removal from the breast [33].

Collectively these results suggest rapid stimulation patterns, stronger vacuums, warmth, and comfort improve efficacy of milk removal by both the infant and the breast pump.
**Effectiveness of milk removal from the breast is enhanced by:**Application of vacuumStrength of vacuumWarmthComfortShield size and design (expression)Double pumping (expression)

## 10. Sucking Swallowing and Breathing

Infant sucking is the primary means of receiving nutrition, soothes the infant and provides the first oromotor exploration experiences [125].

Sucking requires a complex combination of rhythmic movements of the jaw, hyoid bone, lips, tongue, soft palate, to coordinate with swallowing and breathing. Key anatomical differences exist between the infant and adult oral cavity to facilitate breastfeeding. The infant’s feeding method impacts growth and development of the jaw and facial structures, with breastfed infants developing less non-nutritive sucking habits and functional disorders such as mouth breathing or atypical swallowing when compared to bottle fed infants [126,127,128].

## 11. Dynamics of Breastfeeding

For many years it was believed that the primary driver of milk removal from the breast was positive pressure with the infant exerting a peristaltic tongue movement to squeeze milk from the ducts, with additional pressure applied by the mandible. When a sufficient bolus volume collected in the oral cavity the medial tongue then moved the milk bolus to the oral pharynx using a backward-moving wave like motion. The premise for this theory was based on the existence of lactiferous sinuses that stored substantial volumes of milk. More recent evidence has shown that lactiferous sinuses are absent and that the main milk ducts do not in fact store large volumes of milk. Rather, the ME reflex is critical to transporting milk from the alveoli to the ducts making it available for effective milk removal [123,124,129].

**Figure 6:**
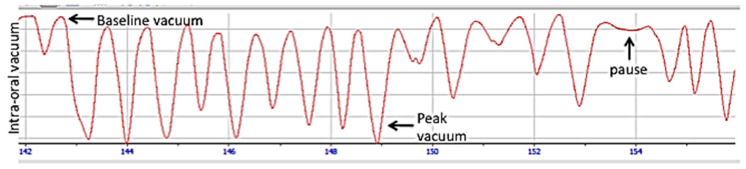
Cyclic vacuum created by a breastfeeding infant. Baseline vacuum is the vacuum required to hold the nipple in place, elongate the nipple and seal to the breast. Peak vacuum is the strongest vacuum created by lowering the tongue during breastfeeding (Geddes et al, unpublished).

**Figure 7:**
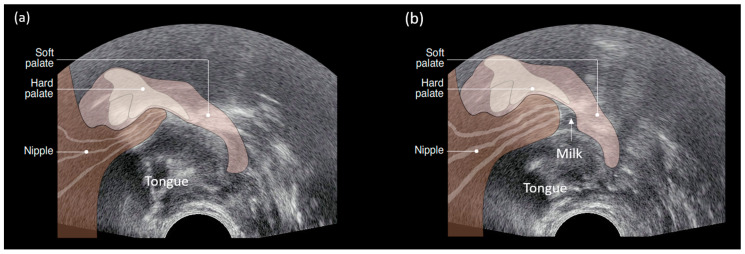
Ultrasound images of the infant oral cavity during breastfeeding (**a**) Tongue up position during breastfeeding. (**b**) Tongue down is drawn down to create a vacuum and milk flows into the oral cavity. ©Medela AG 2006. Used with permission.

Seminal studies conducted in our lab that have simultaneously measured intra-oral vacuum and provided real time ultrasound imaging of the infant tongue during breastfeeding have shown that the application of vacuum is critical to milk removal from the breast. The studies have shown that the breastfeeding infant places the mouth over the nipple and areola to create a seal and enable attachment to the breast. The infant applies a baseline vacuum (−64 ± 45 mmHg) with the tongue resting in apposition with the nipple and soft and hard palate. The application of baseline vacuum elongates the nipple, positioning it close to the nipple hard-soft palate junction (NHSPJ). When the tongue is lowered the posterior tongue and palate move in unison and vacuum strength increases (145 ± 58 mmHg) with the nipple moving towards the NHSPJ (Figure 6). The nipple expands, rendering the nipple ducts visible on ultrasound, and milk flows into the oral cavity that is bounded by the nipple, palate, and tongue. As the tongue elevates, milk ‘slides’ under the soft palate and is cleared from the oral cavity before the tongue returns to the palate. [130,131] (Figure 7). We did not observe wave-like or peristaltic movements of the tongue or squeezing of milk out of the nipple and this has since been confirmed by others [131,132]. The significance of vacuum as the primary mechanism of milk removal has also been demonstrated in studies using an electric breast pump. Mothers who pump at their strongest comfortable vacuum express more milk, more quickly than with weaker vacuum levels [29]. Furthermore, when infants were fed expressed breast milk from a vacuum release teat, they used a tongue motion similar to breastfeeding and consumed comparable volumes of milk [133]. Of note however the peak and baseline vacuums were weaker than that of breastfeeding most likely due to differences in the structure of the nipple and teat.

Breastfeeding consists of both nutritive (NS) and non-nutritive sucking (NNS). Non-nutritive sucking typically occurs at the beginning of a breastfeed to stimulate a ME and near the end of the breastfeed which may serve to satisfy an infant’s urge to suck and facilitate state regulation and self-comfort [125,134]. These two modes of sucking, NS and NNS, differ in both vacuum characteristics and tongue motion. Interestingly we have shown that mean peak vacuums during NS are stronger (−100 vs. −80 mmHg) than NNS suggesting the infant exerts stronger vacuum to actively remove milk effectively and efficiently [135].

Over the duration of a breastfeed we have found that peak vacuums do not change but baseline vacuums become stronger. In addition, sucking frequency for NNS (104 ± 21 sucks/min) is faster than for NS (89 ± 19 sucks/min) [135]. In contrast, sucking rates on artificial teats are markedly faster for NS (120 sucks/min) but slower for NS (60 sucks/min) [134]. The difference is important since it is often assumed that breastfeeding infants should exhibit a slow rhythmic suck when in reality they can vary both their sucking rate as well as peak and baseline vacuums from one suck burst to the next [136]. Furthermore, NNS suck burst duration is roughly half that of NS (median 4.5 s vs. 7.0 s) and may be readily apparent on observation of a breastfeed [135].

### Suck Swallow Breath Co-Ordination

**Figure 8:**
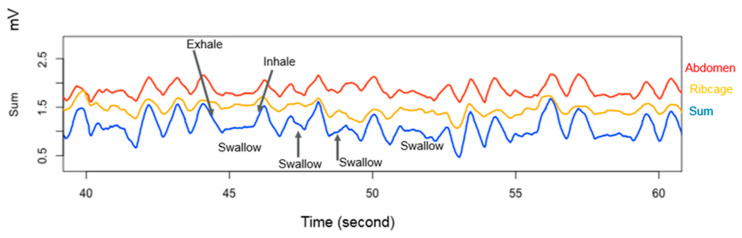
Respiratory traces of a breastfeeding infant using respiratory inductive plethysmography. Signals are recorded from the ribcage (**yellow**) and abdomen (**red**) and the sum of the two is calculated (**blue**). Absence of signal reflects a swallow, downward slope an exhale and upward stope an inhale. Geddes et al., unpublished.

Efficient and effective feeding is reliant on perfectly timed coordination of sucking, swallowing and breathing (SSwB) [137]. It has long been purported that a SSwB ratio of 1:1:1 to 2:1:1 is optimal [138]; however, simultaneous measurement of SSwB during breastfeeding has shown that this is not the case with SSwB ratios within a breastfeed displaying enormous variability ranging from 1:1:1 to 12:1:4 during NS [139] (Figure 8). Thus suck-swallow ratios, a common clinical observation, can change within a breastfeed in contrast to bottle feeding ratios that are typically more consistent [140]. Similar variation of suck:breathe and breathe:swallow ratios have been observed and maybe due to individual patterns of ME suggesting the infants adapts to changing flow rates during breastfeeding [135].

Maturation of SSwB ratios have been shown to change with infant age and development previously [140]. It is likely, however, that milk flow rates and volume increase rapidly after secretory activation driving the change in sucking and SSwB patterns. However, once lactation is established, contrary to common belief, we have not found SSwB ratios to differ from after milk production has been established. The only change we did detect was longer nutritive suck bursts which were comprised of a greater number of sucks, swallows and breaths [135].

These comprehensive results also draw attention to clinical signs of swallowing which consist of observations of long ‘draws’ during sucking (jaw excursion) accompanied by audible swallows [141]. These clinical signs are unreliable and have not been validated by measurements such as test weights to determine the volume of milk removed during a breastfeed [142,143].
**Milk removal by breastfeeding infants**Tongue motion is one of a ‘piston-like’ motion that expands and compresses the nipple evenlyThe infant generates a ‘baseline’ vacuum that draws the nipple and breast into the mouth creating a seal prior to suckingDrawing the tongue downwards generates stronger vacuum to draw the milk from the breastMoving the tongue upwards reduces the strength of vacuum, compresses the nipple, and stops milk flow**Coordination of sucking, swallowing, and breathing by breastfeeding infants**Ratios of sucking, swallowing, and breathing are not consistent nor rhythmic in most breastfeeding infantsSuck-swallow-breath ratios likely change in response to changing milk flow rates during milk ejectionSuck-swallow-breath ratios differ between nutritive and non-nutritive suckingObservation of breastfeeding does not reflect milk removal volumes or patterns

## 12. Nipple Pain

While intra-oral vacuum is important to milk removal, excessive intra-oral vacuum has been associated with nipple pain. More than 60 years ago Gunther reported a sucking intra-oral vacuum up to −200 mmHg in a 2 day-old infant that was associated with nipple pain and damage [144]. Recently, McClellan et al. [145] reported that infants of mothers with persistent nipple pain exerted stronger mean baseline (pain: −91 mmHg; no pain −51 mmHg) and peak intra-oral vacuums (pain: −214 mmHg; no pain: −153 mmHg). In addition, ultrasound analysis showed reduced tongue movement particularly at the base of the nipple, resulting in less nipple expansion [117]. Interestingly, positioning of the nipple with respect to the NHSPJ did not differ in women experiencing pain suggesting increased breast tissue in the infant’s mouth may not be necessarily beneficial. Indeed, we have reported a case study of a woman whose infant exerted very strong mean vacuums (Baseline vacuum: −151 mmHg; Peak vacuum: −233 mmHg) that were not ameliorated by a nipple shield (Baseline vacuum: −41 mmHg; Peak vacuum: −338 mmHg). Further, lower milk transfer volumes were observed in infants of mothers with nipple pain (Pain: 42 ± 31 mL; Control:71 ± 31 mL) [146] although it is possible to establish a full milk supply with assistance [147].

## 13. Nipple Shields

Major advances have been made in the understanding breastfeeding and lactation, sucking dynamics and milk transfer however women experiencing nipple pain remain understudied despite it being a major cause of early cessation of breastfeeding [148]. Various causes of nipple pain have been identified yet treatment options are limited. Nipple shields can improve comfort allowing continued breastfeeding [149] however, the impact of nipple shield use on milk transfer and production is controversial amongst clinicians [150].

Negative consequences of nipple shield use are based on limited evidence of reduced milk transfer in term breastfeeding dyads [151] and with shields that are no longer available [151,152]. In contrast, a pilot study (*n* = 5) found use of an ultra-thin silicone nipple shield found no difference in infant milk intake with and without the nipple shield [153]. We have confirmed this in women with persistent nipple pain, showing no reduction in milk transfer (no shield: 46 mL; shield: 40 mL) nor effectiveness of breast emptying (PAMR; no shield: 59%; shield 53%) suggesting these infants adapted to the shield and oxytocin release was not impeded, such that adequate volumes were removed from the breast to maintain milk production [154]. Importantly in cases of low milk supply, for which we found incidences of about 35%, these were not associated with levels of pain [147] therefore other causes of low milk production such as nipple shield sizing (small shields may compress nipple ducts), delayed secretory activation, no breast changes during pregnancy, pregnancy complications, repeated mastitis, infant hospitalization and maternal depression should considered.

The use of nipple shields did not impact infant sucking dynamics either with infants displaying similar proportions of the feed in NS and NNS with and without a nipple shield (NS: shield: 51%; no shield: 58%; NNS: shield: 11%; no shield: 15%). These similarities may be due to introduction of the nipple shield in the first postnatal week when early imprinting occurs via communication of somatosensory information from Merkel cells [155] in the oral cavity to the cerebral cortex [156,157].

Both nipple pain and the subsequent us of a nipple shield use are often assumed causes of low milk production. The intensity of nipple pain experienced by women during breastfeeding varies throughout the day and over time [145]. We found an average reduction in pain of 25% (by McGill pain questionnaire) with use of a nipple shield [158], without changes in the visual analogue scale scores suggesting the McGill pain questionnaire may be more sensitive to the nuances and complexities of nipple pain [145]. Indeed persistent nipple pain is multifactorial and includes predisposing, cognitive and emotional factors and external influences factors as defined in the Breastfeeding Pain Reasoning Model complicating its assessment [154]. Importantly and in contrast to assumptions, Chertok et al. observed that 90% of women who initiated nipple shield use in the first postnatal week reported a positive experience, and two thirds of women stated that nipple shield use prevented them from stopping breastfeeding early [159]. Indeed, this data is consistent with maternal reports of the nipple shield being instrumental in continuing breastfeeding when experiencing nipple pain/trauma in the early postnatal period.

## 14. Ankyloglossia

We have found that tongue movement differs in infants with anterior ankyloglossia (tongue tie) in that they either appear to compress the tip or the base of the nipple during a suck cycle. This resolved to a more ‘normal’ motion post frenotomy with maternal pain decreasing during breastfeeding and sucking efficiency increasing (mL/min) [104]. As frenotomy rates have increased so has controversy around definitions of tongue tie, whether it impacts breastfeeding and who should be treated. Landmark research examining the anatomy and histology of the lingual frenulum conducted by Mills et al., has been the impetus for rethinking how to approach tongue-tie and the decision to perform a frenotomy. Major findings of these dissections have shown that the frenulum is not a ‘band’ or ‘string’ but rather is a dynamic structure comprised of a midline fold of fascia that inserts into the internal mandibular arc, creating a diaphragm-like structure spanning the base of the mouth. Further, genioglossus is suspended from the floor of mouth fascia, and can be drawn up into the frenulum fold. In addition, the lingual nerve is located superficially, immediately below the fascia, on the ventral surface of the tongue, making both genioglossus and the lingual nerve vulnerable to injury during frenotomy [110,111]. Finally, the frenulum contains Type III collagen fibres which are much more distensible and mobile than the stronger less distensible Type I fibres previously identified [109,160]. Combined, these findings call for tongue tie grading to embrace normal variability and sheds light on concerns of adverse effects of frenotomy providing the basis for more prospective studies [107].

## 15. Preterm Infants

It is now well accepted HM is critical to the health and development of the preterm infant (born <37 completed weeks gestation). However preterm infants typically are not born with the sucking skills required to adequately remove milk from the breast. Infants <34 weeks corrected gestational age may receive all their milk feeds via an intragastric tube and gradually transition to oral feeds as their sucking skills mature. In neonatal nursery settings where mothers cannot stay with their infants, bottle feeds may be offered when the mother is not available to breastfeed.

In the clinical setting preterm milk transfer is usually assessed and estimated through observation of factors such as sustained latch, audible swallows, and number of suck bursts per feed. However, when compared to test weighing, we have shown that the subjective clinical estimates of milk transfer are inaccurate across a range of milk transfer volumes [48] (Table 3). Of critical importance is that we found that preterm milk transfer is typically much lower than the prescribed feed volume, and the absence of any milk transfer is not uncommon [48,116].

Milk transfer is dependent on several maternal and infant factors that cannot be determined through visual observation. Maternal milk supply and the degree of fullness of the breast determine the availability of milk during a breastfeed [161] and the infant’s suck bursts must coordinate with a mother’s ME for the infant to transfer milk [130].

Our seminal research in breastfeeding dynamics has provided the tools to measure and better understand the sucking characteristics of preterm infants. Geddes’ cross-sectional study of 40 preterm infants born <34 weeks gestation and corrected gestational age 32.7–39.9 weeks showed that while preterm breastfeeding infants use a similar sucking action to that of term infants, there are several differences in sucking characteristics. Most notably, when compared to the mean intraoral vacuum of term breastfeeding infants (−114 ± 50 mmHg [130]) we found the intraoral vacuum of preterm infants was less than half (−41 ± 28 mmHg, [116]). Similarly, the proportion of the feed time that the preterm infant spent sucking (38% ± 18%, [162]) was much lower than that of term infants (82%, [135]). Both the weaker intraoral vacuum and reduced sucking time result in a lower sucking efficiency [116] and likely contribute to low milk transfer volumes in preterm infants.

Nipple shields are routinely used with preterm infants to aid sustained attachment and facilitate milk transfer [163]. Our sonographic measurements of maternal nipple diameters for dyads with and without nipple shield use show that nipple diameters are larger with nipple shield use [116], suggesting the infant can expend less energy attaching to the breast (i.e., extending the nipple and holding it close to the NHSPJ). This may in part explain the higher milk transfer volume noted when preterm dyads use a nipple shield. Additionally, the lower vacuums applied by the preterm infant suggests that when using a shield, a reasonably full breast would increase the likelihood of greater milk transfer.

We have also shown that when preterm infants are fed with a vacuum release teat, they are able to remove sufficient volumes of milk using a similar tongue motion as breastfeeding [164]. In addition, they were discharged earlier [165] and had higher rates of exclusive breast milk feeding at discharge from hospital and breastfeeding at 3 months corrected gestational age [166].

Preterm infants have been shown to have a shorter breastfeeding duration than that of their term counterparts [167]. Early weaning is observed despite a strong maternal desire to breastfeed with intended durations similar to that of mothers of term infants [47]. Our studies indicate that the greatest barrier to continued breastfeeding beyond discharge from the neonatal unit is insufficient milk supply, with few other breastfeeding difficulties cited [47,168]. Mothers that were unable to produce enough milk to meet their infant’s needs at the time of discharge from hospital were at high risk of early weaning [168]. While there are concerns about reduced milk transfer with nipple shield use, we found that nipple shield use at 2 weeks corrected gestational age did not impact breastfeeding to 3 months corrected gestational age [168]. Our group’s involvement in studies of secretory activation, milk synthesis and expression intervals in mothers of preterm infants [42,102], as well as current investigation of the effect of pregnancy complications on secretory activation and subsequent milk production will contribute to the early management of lactation after preterm birth.

Factors influencing breastfeeding and milk removal are summarised in Table 4.

## 16. Influences of Maternal Body Composition on Milk Composition

Human milk promotes optimum growth, development, and health of the infant; however the mechanisms that govern both the variation of composition and the pathways by which it delivers benefits to the infant are not well understood [169]. Increasingly maternal and environmental factors are being associated with milk composition. A recent systematic review indicated maternal adiposity was related to HM lactose and fat concentrations. However our study failed to confirm these relationships HM [170,171]. We have conducted several studies, to determine relationships between maternal body composition (BC; lean and fat mass) and HM composition. We have found that higher maternal BC (%fat mass (FM), body mass index (BMI)) is associated with increased concentration of whole HM leptin [171] but not skim HM leptin, which is lower in leptin content [172]. This is consistent with the only other study measuring whole HM leptin using pre-pregnancy BMI as a measure of adiposity confirming maternal BC may influence HM leptin [173,174]. Variation of reported relationships between maternal adiposity and leptin depend on methods of measurement and whether skim or whole milk was analysed as well as number of study participants. For example, skim HM leptin concentrations in obese mothers (*n* = 50) were double of that of normal-weight mothers (*n* = 50) [175] whereas no differences for whole HM leptin was found in our smaller longitudinal cohort [176]. Therefore, future analysis for leptin should be conducted in whole HM.

We have also found that increased maternal BMI is associated with increased HM cortisol concentration [177]. Indeed individuals with higher BMI have increased circulating cortisol concentrations [178] that could translate to higher concentrations in HM. Concentrations of these hormones in our study were highly variable within and between women and remained constant throughout the first 12 months of lactation, suggesting a more complex relationship between HM glucocorticoids and maternal adiposity.

Like whole HM leptin, higher concentrations of HM protein are associated with increased maternal adiposity and are not identical in all tested cohorts [171,179]. We observed positive associations between total protein and maternal %FM, but not BMI [171]. In a longitudinal cohort, we found positive associations between whey protein concentrations and maternal weight, BMI, fat free mass (FFM), FFM index (FFMI), and FM index (FMI), with %FM showing no associations [179]. As with leptin, there are studies reporting both presence and absence of such associations [170]. To add to the complexity further, we have shown that HM total protein concentration does not change day-to-day [180], week-to-week [181] or during a short maternal dietary intervention [182].

Increasingly it appears that the mother modulates several components in the milk. We have not identified associations of maternal adiposity with lactose or total carbohydrates, nor have we found strong associations with total (estimated) human milk oligosaccharides (HMO) [183]. However, more recently HMOs have been shown to change with a maternal dietary intervention [184], mode of delivery and parity [185]. Studies of HMOs and maternal BMI report conflicting associations [186,187]. Indeed, larger longitudinal studies including measurement of maternal BC are required to clarify associations with HM components.

## 17. Gastric Emptying and Infant Body Composition

Gastric emptying (GE) is a key regulator of appetite [188]. However, there is much to be learned about the programming potential of HM components, how their patterns change throughout the lactation period and their impact on the GE rate of the breastfed infant. Few components, have been investigated in connection with the regulation of infant milk intake and feeding patterns, including pre-feed gastric residual (GR) volumes and GE rates of breasted infants.

The absence of suitable validated techniques for the determination of GE has restricted the evaluation and understanding of GE in the breastfed infant as a mechanism of appetite control. We have validated an ultrasound technique to assess GE in a preterm population [189]. We have also shown that HM components are associated with GE and therefore, potentially impact infant appetite regulation and BC, evidenced by relationships between feeding frequency [190,191]; and associations between feeding frequency, milk intake and infant BC [192].

Measurements of infant BC have been largely limited to anthropometric measurements such as birthweight, weight, length, head circumference and BMI due to limited access to comprehensive BC measurement techniques. Recently emphasis has been drawn to the quality, not just the quantity of growth in the context of childhood obesity. For example preterm infant BC in early life appears to play an important role in programming long-term health outcomes including obesity and other non-communicable diseases [193]. As such adult BC measurement techniques are being adapted and to the paediatric population, and include bioelectrical impedance spectroscopy (BIS), dual X-ray absorptiometry (DXA), isotope dilution, magnetic resonance imaging (MRI), ultrasound, whole-body air-displacement plethysmography (ADP), computed tomography (CT) and others [194].

## 18. Preterm Infants

Using our validated ultrasound technique in preterm infants [195] we found that GE of pasteurised donor human milk (PDHM) was slower than mother’s own milk (MOM) both during feed delivery and the postprandial period [196]. Immediately after feeding, the volume of PDHM retained in the stomach was 23% higher than MOM, reducing by 15%/30-min until the next feed (3 h). However, the rate of emptying of PDHM is closer to that of MOM than infant formula, with calculations indicating that postprandial retained proportions of formula are 23% to 29% higher than that of MOM [197,198]. Final gastric residual volumes (FGR) were not different between milk types indicating that the same end point was met for both PDHM and MOM indicating the slower emptying of PDHM is not detrimental. Further gastric residuals were 12% to 22% of the feed volume, which is below the 30% to 50% considered a marker of feeding intolerance [199] and supports clinical observations and evidence that PDHM is unlikely to be implicated in large FGRs associated with feeding intolerance [200].

Interestingly compositional differences between PDHM and MOM did not explain the disparity in GE observed in our study. Whilst higher MOM casein and lactose concentrations are associated with faster emptying [201] feeds of PDHM emptied more slowly than MOM despite having higher concentrations of casein and lactose. It is possible that the effect of pasteurization or frozen storage alters components of donor HM and so counters the effects of casein and lactose on emptying. Holder pasteurization also causes complete inactivation of the bile salt stimulated lipase (BSSL) and lipoproteinlipase [202]. Bile salt stimulated lipase enables almost complete hydrolysis of HM triacylglycerol through its synergistic action with pancreatic lipase-related protein 2 [203]. As the presence of lipids in the duodenum and ileum slows GE through triggering of the ileal brake, it is possible that restricted lipolysis of pasteurized milk is a mechanism for slower emptying of PDHM [204]. Also, heat treatment of bovine milk has been shown to alter the structure of whey proteins resulting in interactions between denatured whey proteins and casein micelles [205]. It is therefore conceivable that Holder pasteurization creates similar interactions in HM possibly negating the faster GE associated with higher human casein concentrations.

We found no evidence to support a difference in GE according to infant sex. We did detect faster GE during feed delivery in male infants however this was explained by the lactose concentrations of PDHM fed to male infants that were on average 6.1 g/L and 14.2 g/L higher than that of PDHM and MOM fed to females [196].

There is limited evidence of a relationship between energy concentration and GE in preterm infants. Our studies show that the energy concentration of HM feeds ranging from 12.6–30.4 kcal/30 mL did not influence GE, likely because triacylglycerol, the predominant HM lipid that contributes to HM energy density, differs from other lipid sources in that it does not trigger the ileal brake to slow GE [197,206,207] (refs). The preterm infant’s diminished GE response to increasing lipid and energy concentrations may serve to facilitate the physiological emptying of fat-rich HM that is ingested towards the end of a breastfeed [65]. Our GE rates are similar to those observed for MOM and formula feeds of 5–20 kcal/30 mL [208]. In contrast, an older study reported increasing energy concentrations were associated with slower postprandial emptying for formula feeds of 5–20 kcal/30 mL from 20 min post feed, with differences between 20 kcal and 24 kcal noted only at 80 min post feed [209]. The study did not account for the osmolality or specific nutrient concentrations of the bovine-based formula feeds which differ from HM and are known to influence gastric emptying [206].

Our ultrasound technique also allows assessment of the effect of the addition of human milk fortifier (HMF) to HM feeds on GE. When bovine-based HMF was added to achieve an assumed caloric density of 24 cal/30 mL, slower GE was observed across all 30-min time points between feeds [196]. The magnitude of the effect was smaller for PDHM than for MOM, possibly due to differing interactions/digestion of HMF between MOM and PDHM due to the degradation of several HM bioactive factors by heat pasteurization. This finding concurs with Ewer & Yu who reported an average gastric half emptying time of fortified MOM to be 48 min, more than double the 21 min average for unfortified MOM [210].

Supine infant positioning was associated with slower GE with retained feed proportions on average 16% higher than that of infants positioned prone or right lateral across the postprandial period [196]. The observed inter-individual effects of positioning on GE are consistent with several published studies [211,212] and further support the use of prone positioning as a conservative clinical management strategy for feeding intolerance in the neonatal unit setting.

A gastric residual volume ≤2.5 mL that is predominantly curd appears to be usual for stable preterm infants [213], with most (62%) 3 hourly feeds resulting in empty stomachs. This finding raises the possibility that feeding of 3 hourly volumes may be tolerated if fed every 2.5 h. The gastric residual, as a proportion of the feed volume, was similar between MOM and PDHM and were 10% and 15% for unfortified and fortified feeds respectively. These are similar to the 15% and 16% residual feed proportions reported for MOM and infant formula, respectively [207,214] and below the 30%−50% considered to be a sign of feeding intolerance [199].

Sonographic examination of gastric contents after HM feeds showed that the presence of curding, and its echogenicity, or density, were similar for MOM and PDHM. Immediate post feed ratings of high curd density and high curd volume were associated with higher HM casein concentration, and HMF also significantly contributed to high curd volume [189]. As neither of the two HMF products used in the study contained casein, it is likely the higher curd volume and density associated with HMF resulted from a more acidic environment caused by HMF that facilitates HM protein precipitation [215]. The presence of small curd volume in the stomach suggests there is continued delivery of nutrients to the ileum beyond emptying of the liquid portion of a human milk feed.

Our gastric emptying studies confirm that HM is well tolerated by the preterm infant; despite changing composition over time and between women, no clinically significant impact on gastric emptying is observed. Current heat treatment of donor HM and commercial HMF products slow GE somewhat but not to the degree that causes feeding intolerance in medically stable preterm infants. Further, with complete emptying of 3-hourly feed volumes typical for many infants, it may be possible to explore more frequent feeding of similar volumes when additional caloric intake is indicated.

Infants born preterm are susceptible to postnatal growth restriction and will benefit from aggressive nutritional management in order to achieve optimal growth and BC parameters. Body composition measurements in preterm infants are difficult to obtain due to compromised health and physical limitations such as their fragile skin that prevents the use of callipers, the need to avoid frequent blood collection (e.g., tracer dilution techniques) and increased susceptibility to radiation. They are also relatively overhydrated, with higher body water content compared to children and adults, leading to overestimation of FFM when using bioelectrical impedance analysis [216]. This leaves few methods that are suitable for the hospitalised preterm infant with limitations due to the risks of overhandling, infection, and thermal instability. Previous studies have reported lower FFM, and higher FM compared with term infants at hospital discharge, thus increasing their risk of developmental issues, metabolic syndrome, and obesity later in life [217,218,219].

We have found, with air displacement plethysmography that there were relationships between milk composition and changes in BC preterm infants fed fortified HM. Increased fat and total energy intakes were associated with increasing FM whilst increasing protein intake when considered with carbohydrate intake was associated with increasing FFM [220]. We have also established that ultrasound is sensitive enough to detect HM macronutrient-related changes in accrued adipose and muscle tissue measured at multiple sites: abdomen, scapula, mid-thigh and mid-arm in preterm infants [221]. Enteral volumes (predominately HM) were positively associated with adipose to muscle ratio whilst timing of fortification, carbohydrate intake and the protein energy ratio of intakes moderated ratio of the adipose to muscle tissue accretion in preterm infants. This study demonstrated that ultrasound may offer a clinically useful tool to obtain non-invasive obtain serial infant BC measurements.
**Gastric emptying of preterm infants**Gastric emptying rate of pasteurised donor human milk (PDHM) is more similar to mothers’ own milk (MOM) than infant formulaFinal gastric residual volumes for PDHM and MOM are similarFinal gastric residual volumes for PDHM and MOM at 12–22%Human milk casein and lactose impact gastric emptyingGastric emptying does not differ by infant sexFortification of MOM and PDHM slows gastric emptyingSupine position of infant slows gastric emptyingMOM and PDHM produce similar curding patterns**Body composition of preterm infants**Increased fat and total energy intakes are related to increasing fat massIncreasing protein intake when considered with carbohydrate intake is related to increasing fat-free massEnteral volumes are positively associated with adipose to muscle ratioTiming of fortification, carbohydrate intake and protein energy ratio of intakes moderate ratio of the adipose to muscle tissue accretion

## 19. Term Infants

There is evidence of differences in GE between infants fed infant formula compared to those fed HM, yet breastfed infants have not been studied in depth with respect to milk composition. We have investigated the effects of multiple factors, including appetite hormones, macronutrients, BC and maternal factors, on GE.

We found higher feed volumes were associated with faster GE rate, higher post-feed stomach volumes, and longer GE times. Greater feed volumes were also associated with smaller residual volumes prior to the feed and larger residual volumes prior to the next feed. Importantly, during exclusive breastfeeding period we found no effect of infant age or sex on feed volume, GE or breastfeeding patterns, but larger (not older) infants with higher adiposity had longer time between feeds, indicating a potential link between feeding frequency, GE, and BC. It also implies that feeding frequency is dictated by an individual’s growth rate and development rather than age, providing further support for breastfeeding on demand for the duration of the breastfeeding relationship.

## 20. Proteins

**Figure 9:**
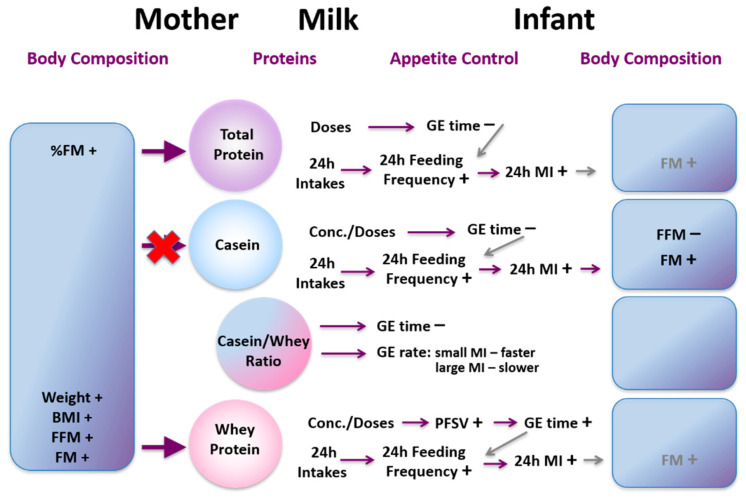
Possible pathways of lactocrine programming of the infant by the human milk proteins. BMI—body mass index; CDI—calculated daily intakes; Conc.—concentrations; doses—amounts of human milk component ingested during a single breastfeed; FFM—fat-free mass; FM—fat mass; %FM—percentage fat mass; GE—gastric emptying; MI—milk intake; PFSVs—post-feed stomach volumes; − negative association; + positive association. Purple arrows indicate the direct associations between components and infant BC. In the case where direct relationships between infant body composition and human milk components are supported by the relationships of human milk components with infant gastric emptying factors and breastfeeding parameters, these relationships have been included and could be integrated into possible pathway. Grey arrows indicate further possible pathways, although no direct association of component unit with infant body composition has been established (or not analysed in case of doses).

We have found that specific protein composition rather than total protein appears to influence GE either indirectly or directly. For example, the casein:whey ratio modified the GE rate depending on the volume of milk consumed, such that higher casein:whey ratios were associated with faster GE of small feed volumes and a slower GE rate of larger feed volumes [190]. Furthermore, higher casein concentrations and intakes were associated with shorter GE time, which may result in higher feeding frequency, and in turn, higher 24 h milk intake and infant adiposity (Figure 9). In this cohort the association between feeding frequency and infant adiposity strengthened with duration of lactation, consistent with Ay et al. [222]. In contrast to casein, higher whey protein concentrations and intakes were associated with longer GE time [190], which may result in lower milk intake and reduced adiposity [192] (Figure 9). Thus, both feeding frequency and GE are in part likely regulated by HM casein and whey concentration. However, it was not the casein concentration but daily intake of casein that was directly associated with infant BC; negatively with lean mass and positively with FM with associations strengthening in the later months of lactation [179]. This suggests that higher daily doses of casein may down-regulate the accrual of lean body mass in infants, potentially by decreasing time between feeds and increasing the volume of milk consumed. These results further clarify role of the HM protein as a potent appetite regulator and draw attention to the importance of the HM protein composition.

## 21. Immune Factors

**Figure 10:**
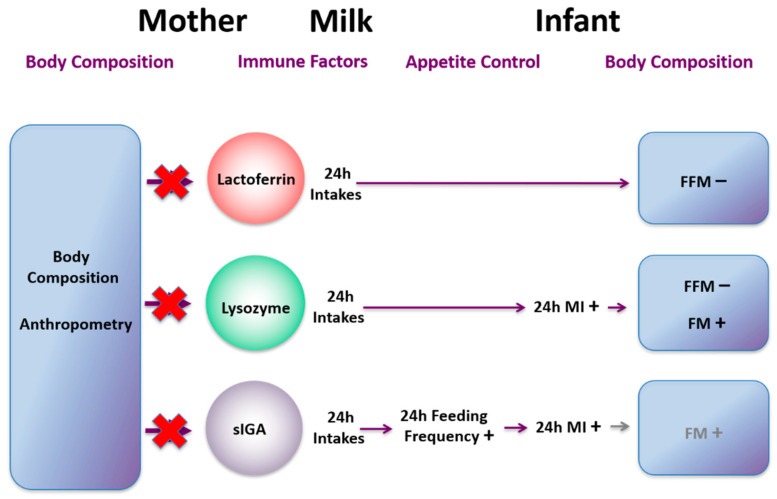
Possible pathways of lactocrine programming of the infant by the human milk immune factors. CDI—calculated daily intakes; FFM—fat-free mass; FM—fat mass; sIgA—secretory immunoglobulin A; − negative association; + positive association. Purple arrows indicate the direct associations between components and infant BC. In the case where direct relationships between infant body composition and human milk components are supported by the relationships of human milk components with infant breastfeeding parameters, these relationships have been included and could be integrated into possible pathway. Grey arrow indicates further possible pathway, although no direct association of component unit with infant body composition has been established.

HM contains multiple immunological factors that provide protection against various health challenges [223,224,225]. Within the whey fraction, lactoferrin, lysozyme and sIgA are present in high concentrations, potentially indicating multiple roles in infant programming [226,227]. We have shown that these bioactive components relate positively to breastfeeding frequency (sIgA) and milk intake (lysozyme, sIgA), and that these relationships extend further to infant BC [228] (Figure 10). Higher lactoferrin intake was associated with lower infant FFM index whilst higher lysozyme intake related to higher infant adiposity. Although not associated with maternal BC, the concentrations of these components continued to increase throughout the first year of lactation, ensuring the same level of infant protection despite the reduction in milk intake.

Lactoferrin is being explored as clinical intervention for infant health [229], and for the first time we have evaluated how natural variations of this HM component relate to BC of term breastfed infants. The observed negative relationship of lactoferrin intake with infant lean mass could be explained by modulation of infant gut microbiome [230], as the gut microbiome has been implicated in infant weight gain and obesity [231], as well as by increasing the bioavailability of iron to the infant. Iron supplementation in iron replete infants has been shown both to decrease linear growth and weight [232]. The exact mechanisms of these outcomes are not clear and interactions between lactoferrin and infant growth appear complex requiring further study.

Lysozyme is important not only for the immune protection of the infant but for infant growth and now BC as we have shown a positive relationship with FM and negative with FFM [228]. Preterm infants have exhibited better growth and improved gastrointestinal function when administered lysozyme [233]. The effects of HM lysozyme may be mediated through improvement of infant gut health and optimized digestion, resulting in increased absorption of nutrients and subsequently, increased fat accretion. Or alternatively, that lysozyme, not unlike leptin, also enhances innate and adaptive immune responses [234], therefore lysozyme may also be implicated in the two-way relationship between the obesity and immune status.

Whilst HM sIgA demonstrated no strong associations with infant BC, daily intake was positively associated with breastfeeding frequency which in turn is also related to 24 h milk intake (Figure 10), potentially affecting infant adiposity. Like lysozyme, broad spectrum of sIgA antibodies are implicated in the development of infant mucosa and anti-inflammatory and tissue protective activities [235]. These findings warrant further investigation of other immunological factors of HM in relation to infant growth and BC.

## 22. Appetite Hormones

HM hosts a plethora of hormones that are biologically active, including adipokines and glucocorticoids that may programme appetite [236], and we have found interesting relationships of HM adiponectin with infant GE. Higher concentrations and intakes of adiponectin were associated with longer times between feeds in term breastfed infants [190], which may be implicated in the growth-regulating effect of adiponectin in the first 4–7 months of life, evidenced by high HM adiponectin concentrations being associated with lower infant weight and FFM [176]. Extended GE times may culminate in fewer feeds per day and potentially lower 24-h milk intake, restricting both volume and energy to the infant and slowing growth. This hypothesis is further supported by our longitudinal study of infant BC that showed that higher HM adiponectin intake being associated with lower infant lean mass and increased adiposity over the first year of life [176].

**Figure 11:**
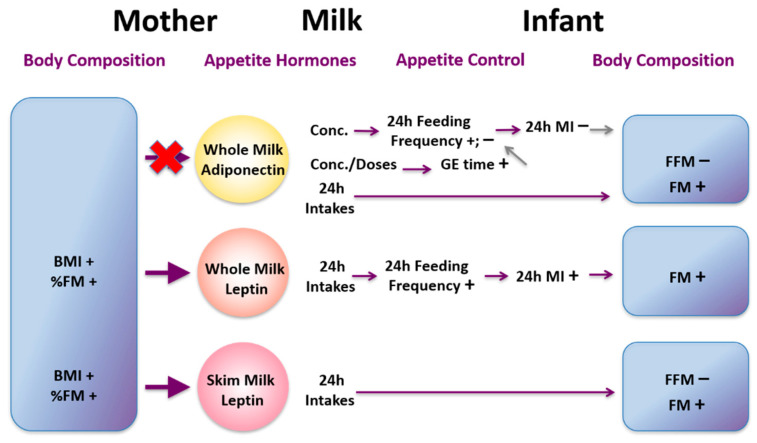
Possible pathways of lactocrine programming of the infant by the human milk appetite hormones. BMI—body mass index; CDI—calculated daily intakes; Conc.—concentrations; doses—amounts of human milk component ingested during a single breastfeed; FFM—fat-free mass; FM—fat mass; %FM—percentage fat mass; GE—gastric emptying; MI—milk intake; − negative association; + positive association. Purple arrows indicate the direct associations between components and infant BC. In the case where direct relationships between infant body composition and human milk components are supported by the relationships of human milk components and infant gastric emptying factors and breastfeeding parameters, these relationships have been included and could be integrated into possible pathway. Grey arrows indicate further possible pathway, although no direct association of component unit with infant body composition has been established.

Whole HM leptin content did not display any associations with GE or breastfeeding parameters [190] (Figure 11), indicating that long-term effects of leptin in human infants are possibly stronger than the potential short-term satiety effects observed in rodent models. This also highlights the difficulty of extrapolating results in animal models to humans [237]. However, HM leptin intake is related to infant feeding frequency and BC development, with higher daily intake of both, whole and skim milk leptin associating with greater deposition of adipose tissue [176]. Skim milk, however, is not representative of what infant ingests and results for skim milk leptin should be interpreted with caution.

## 23. Glucocorticoids

HM contains the glucocorticoids, cortisol, and cortisone, which are involved in regulation of inflammation and metabolic homeostasis and may impact infant gut maturation and the microbiome [238]. Little is known about how these hormones affect infant growth and development of BC. For the first time we reported that higher concentrations of HM cortisol and cortisol to cortisone ratio are related to greater infant adiposity [177]. Elevated circulatory cortisol is known to be a potent stimulator of body fat mass gain in adults [239,240] and recently in 2-year-olds [241].

**Figure 12:**
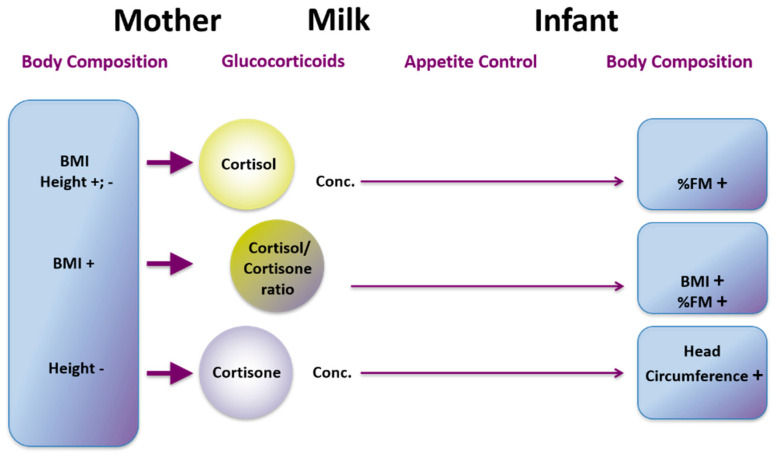
Possible pathways of lactocrine programming of the infant by the human milk glucocorticoids. BMI—body mass index; Conc.—concentrations; %FM—percentage fat mass; − negative association; + positive association. Purple arrows indicate the direct associations between components concentrations and infant BC and anthropometry.

Additionally, cortisol was positively related to head circumference, an important indicator of infant brain size and a proxy for intracranial volume, neurological development and cognitive function [242] all of which are commensurate with breastfeeding [243,244]. The cortisone relationship with head circumference the reverse of that reported for chronic prenatal maternal stress during pregnancy (smaller head circumference) [245] (Figure 12). Whilst underlying mechanisms for these associations are unclear, these findings should be confirmed with daily infant intakes of glucocorticoids in addition to concentrations.

## 24. Carbohydrates

**Figure 13:**
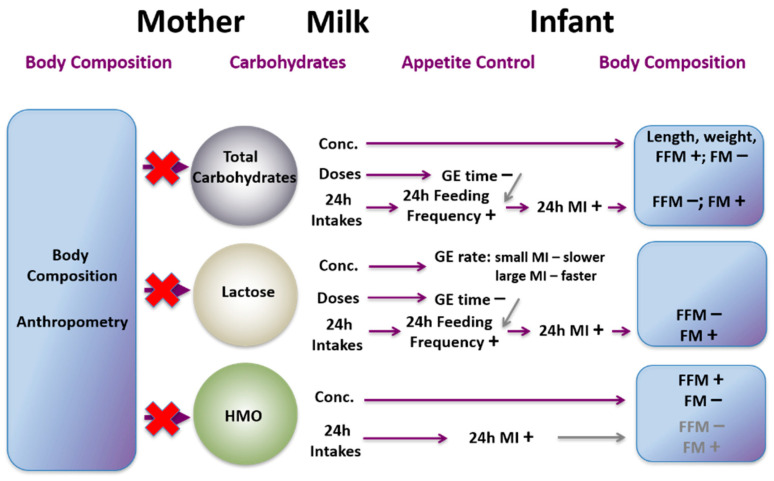
Possible pathways of lactocrine programming of the infant by the human milk carbohydrates. BMI—body mass index; CDI—calculated daily intakes; Conc.—concentrations; doses—amounts of human milk component ingested during a single breastfeed; FFM—fat-free mass; FM—fat mass; GE—gastric emptying; HMO—human milk oligosaccharides; MI—milk intake; − negative association; + positive association. Purple arrows indicate the direct associations between components and infant BC. In the case where direct relationships between infant body composition and human milk components are supported by the relationships of human milk components with infant gastric emptying factors and breastfeeding parameters, these relationships have been included and could be integrated into possible pathway. Grey arrows indicate further possible pathways, although no direct association of component unit with infant body composition has been established (or not analysed in case of doses).

Our research has also provided new insights about relationships of HM carbohydrates with infant GE [190] (Figure 13). We have found that both total carbohydrates and lactose are associated with GE, feeding frequency and infant 24 h milk intake culminating in relationships to adiposity [190] (Figure 13). This highlights the complexity of pathways affected by HM components and that infant intake, as evidenced by our finding that higher lactose concentrations are associated with slower GE rate of small feed volumes and faster GE rate of large feed volumes [190]. These results support the findings of Khan et al. [65] who reported a positive association between lactose concentration and feeding frequency.

We have reported that concentrations and, more importantly, daily intakes of HM carbohydrates are associated with development of infant BC and are differentially related to infant anthropometry and BC [183]. Furthermore, the directions of the associations with infant BC were not uniform for the daily intake and concentration of the same measured carbohydrate. Lactose concentration showed no associations with infant BC, yet lactose intakes were negatively associated with lean mass and positively with FM. Intake of total carbohydrates showed similar relationships with lean and fat mass, but relationships with concentration were reversed with more carbohydrate associating with more lean mass, less adiposity and higher infant length and weight.

Whilst we did not directly measure concentrations of HMO in our cohort, we found that like total carbohydrates, total HMO concentration related positively to infant lean mass and negatively to adiposity, yet intake of HMO displayed no associations. Of note, HMO intake remained the same over 12 months post-partum. Our results support recent findings of differential associations between individual HMO and infant BC [246]. Total HMO on the other hand had no impact on infant GE which was expected as HMOs target the small intestine and have been shown to modulate the gut microbiome therefore, having an indirect effect on BC [247].

Given contrasting relationships of HM component concentrations and intakes, infant intake of components may well reflect better the nutritional physiology of the breastfed infant. These findings also indicate the possibility of intervention via modulation of both milk intake and the infant gut microbiome, which is implicated in adiposity and development of obesity [248].

## 25. Lipids

HM lipid fraction accounts for 50% of the energy content and is thus an important contributor to infant growth. Dose or concentration of HM fat showed no effect on term infant GE rate or time [190], consistent with lack of associations between fat and feeding frequency reported previously [60,65]. Higher HM fat intakes have been associated with lower weight-for-age *z*-scores [249], but also higher weight gain [250]. Higher 24-h fat and total energy intakes have also been associated with higher infant weight and FFM at 3, 6 and 12 months [251]. These studies should be interpreted with caution due to sampling of HM lipid not being straight forward. Total fat increases across a breastfeed and changes throughout the day due to the relationship between fat content and the fullness of the breast [63,252]. As such sampling is problematic with one sample not being representative of either fat content or infant fat intake as illustrated by George et al. [253]. Further, analysis differs according to analytical technique, with the creamatocrit method offering an attractive alternative to more complex biochemical assays [254,255] such that it could be integrated into the clinical practice, particularly in the area of preterm nutrition, to increase the energy content of milk [256].
**Gastric emptying in term infants**Increased feed volumes associate with faster rates of gastric emptying but longer gastric emptying times.Infant sex and age did not relate to gastric emptying characteristicsLarger infants had longer times between feedsCasein:whey ratios influence gastric emptying rate according to volume of the feedInfant intakes of adiponectin are associated with longer times between feedsInfant intake of whole HM leptin is positively associated with feeding frequency in first 5 months of lactation**Body composition of term infants**Infant casein, lactose and total carbohydrate intake has a positive association with infant fat mass and a negative one with infant lean massInfant lactoferrin intake has a negative relationship with infant lean massInfant lysozyme and whole HM leptin intake have a positive relationship with infant fat mass

## 26. Infant Health

HM is much more than the nutrition that supports optimal infant growth. It is a compositionally complex and dynamic fluid that supports both the innate and adaptive infant immune system [257]. Many milk components have dual roles in the nutrition and protection of the infant. For example, lipids are most often regarded as nutrition but there is mounting evidence of their association with health. Using new methods [258] we have recently discovered 98 novel triaclyerglicerides (TAGs) and found that infant intake of palmitic acid-containing and lauric acid-containing TAGs differed between healthy and unwell infants indicating responsiveness of the milk [259]. This is consistent with our previous study that showed reductions in the proportion of capric (C10:0) and lauric acids and increases in palmitoleic and stearic acid [260] in unwell mothers and infants. Indeed it also highlights both the importance of measuring component intake [261] and with respect to total lipid employing extensive sampling [253].

Similarly, we have documented increases in the leukocyte content in response to maternal and/or infant infection. The smallest response was seen if only infant was ill and the greatest with maternal mastitis and upon recovery leukocyte content returned to the health baseline level. This increase in leukocytes was also accompanied by significant changes in lactoferrin, sIgA IgG, and IgM concentrations [262].

These changes in the milk components as responses to maternal and infant health hold promise potential therapies; however this work is hampered by the ability to isolate components and scale up their production [263]. In contrast, modulation of milk via maternal interventions may improve health in an individualised fashion.

## 27. Human Milk Microbiome

**Figure 14:**
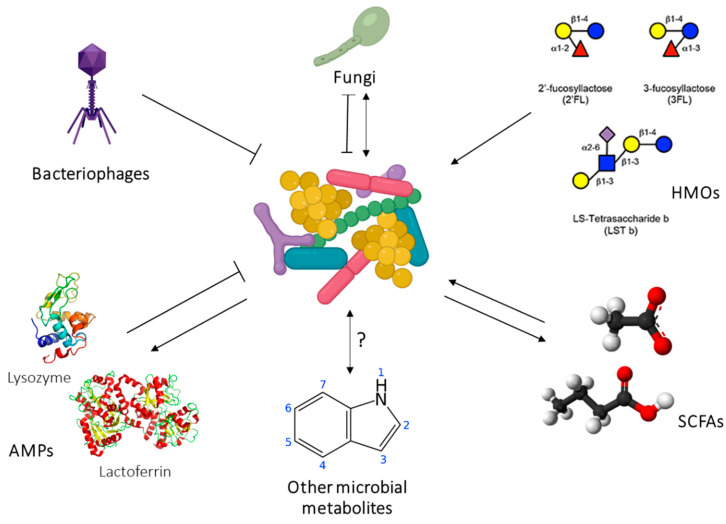
Beyond bacteria: relationships between human milk bacteria and other microbial and non-microbial components of milk. AMPs (antimicrobial proteins) have antibacterial effects, but are also liberated from their parent proteins via the proteolytic action of certain members of the milk microbiome [271]. Bacteriophages in milk can infect bacteria. Milk fungi have been both positively and negative correlated to milk bacteria [272]. HMOs are prebiotics which promote the growth of certain milk bacteria. SCFAs are both a product of a substrate for bacterial metabolism. Other bacterial metabolites such as indoles likely exist in milk. To characterise the influence of the human milk microbiome on infant health, an integrative analysis of these components is required.

The development of the gut microbiome in the first years of life has been strongly associated with immune and metabolic outcomes in large human cohort studies, and in interventional animal models [264]. Importantly, HM shapes the infant microbiome through direct transfer of bacteria as well as bioactive components such as HMOs, antimicrobial proteins, and short chain fatty acids (SCFAs) [265]. The human milk microbiome (HMM) is therefore of great interest as a target for developmental programming of health. However, while the composition of the HMM has been extensively characterised ([265]), little is known about the origins of this community or the host-microbiome interactions at the breast. Host-microbe and microbe-microbe interactions in the lactating mammary gland are likely to be highly complex. Inter-kingdom interactions between human cells, bacteria, fungi, and viruses, as well as interactions involving host-derived and microbe-derived bioactives likely influence mammary gland and infant health (Figure 14). Indeed, it is not even known whether the lactating mammary gland hosts a permanent resident microbiome, or whether bacteria are bought in from exogenous locations (such as the maternal gut or infant oral cavity) and survive temporarily before being swept out of the breast via a ME (Figure 14). Stinson et al. have described these two possibilities as the “mucosal interface model” and the “constant influx model”, respectively [265]. Regardless of whether the bacteria detected in milk are permanent residents or mere “tourists”, numerous HMM taxa have been shown to be vertically transferred from mother to infant via milk [266,267,268,269]. Factors that shape HMM composition, such as maternal diet, may thereby influence infant colonisation dynamics, with implications for infant health. The potential for maternal diet during lactation to influence infant gut microbiome dynamics has been reviewed extensively by Sindi et al. [270]. This evidence, from observational human studies and animal models, paves the way for future intervention studies to assess the impact of maternal diet on infant microbial development.

In addition to live bacteria, HM also contains the products of bacterial metabolism, such as SCFAs. These immunomodulatory metabolites SCFAs (formate, acetate, propionate, butyrate, and valerate) are the end products of bacterial fermentation of fibre in the gut and are transported systemically around the body, including to the lactating mammary gland. They have been shown to elicit a broad range of immunological effects, including promotion of regulatory T cell responses and immune tolerance, synthesis of dendritic cell precursors, and epithelial barrier integrity in the gut [273,274,275]. HM therefore has a two-fold influence on infant health: by directly seeding the infant microbiome with human milk bacteria, and by exposing the infant to bacterial metabolites formed in the maternal gut. Emerging evidence suggests that SCFAs may protect infants from developing atopic disease [273,276]. Our research has shown that milk from atopic mothers contains a significantly reduced concentration of SCFAs compared to that of healthy mothers [277]. This finding may in part explain why breastfeeding does not protect against atopy if the mother herself is atopic [278,279]. HM SCFAs have also been associated with infant BC [280]. They are therefore of interest for both infant growth and infant immune development. Given that maternal SCFA levels may be modulated by diet, these bacterially derived metabolites represent an exciting opportunity for intervention to optimise infant health.

Our group has established methodologies for studying the HMM from collection to analysis. We demonstrated that milk expressed using an electric breast pump does not differ in its bacterial composition to milk expressed by hand [281]. This finding is reassuring for those designing HMM studies. We also assessed four commercial DNA extraction kits for their ability to extract DNA from HM [282]. We found that two of the kits could not reliably extract DNA from HM. Of the two remaining kits, a similar bacterial DNA profile was extracted, but one kit co-extracted a high level of contaminants. Such inter-kit variability may help to explain some of the variation seen in HMM composition between studies. A significant challenge in working with HM is the fat fraction, which interferes with DNA extraction. This fraction is therefore routinely discarded prior to DNA extraction in HMM studies. We demonstrated that this fraction contains bacterial DNA, suggesting that bacteria may be trapped in the lipid layer by milk fat globule membranes [283,284]. However, reassuringly, the fat fraction did not differ in bacterial composition to the cell pellet, suggesting that discarding the fat fraction prior to extraction would not alter the composition of bacteria detected downstream. This is particularly important, as we have shown that inclusion of the fat fraction reduces DNA extraction efficiency by ~40% [283]. By focusing on robust methodologies for reproducible data, we have raised the standards of the field.

## 28. Donor Human Milk

In past decades there has been a resurgence in the establishment of donor milk banks in an effort to provide HM for vulnerable infants [285]. Donor milk has been shown to have positive effects of infant mortality and morbidity [286] whilst being economically cost effective [287]. We have been active in the formulation of best practice in milk banking which varies according to geography and resources [288] as well as investigating pasteurization methods. Thermal pasteurization of milk is almost universal in milk banks however in the process of eliminating bacteria and most viruses [289,290] bioactivity is often dramatically reduced [291]. Reduction in the loss of bioactivity can be altered by reduction in pasteurization temperature [292], combinations of time and temperature, as well as other technologies [291,293]. Our group has pioneered UV-C treatment [294] in an effort to preserve the bioactivity of protective components in milk. It shows great promise in eliminating bacteria [295] including cytomegalovirus [296] not at the expense of immune protein activity [297]. More recently we investigated the heat stable enterotoxin produced by the potential pathogen S. aureus. S. aureus enterotoxins are linked to gastritis and necrotizing enterocolitis. Spike in experiments (S. aureus, and enterotoxin) at various storage temperatures and times confirmed a rapid decline in both the bacteria and enterotoxin in raw and UV-C treated milk providing more evidence of promise for this method of processing donor milk [298,299].

## 29. Human Milk Cellular Content

HM cell studies offer insight into the biosynthesis pathways involved in milk secretion, the microenvironment of the lactating mammary gland and the potential physiological role of HM cells for the developing infant.

**Figure 15:**
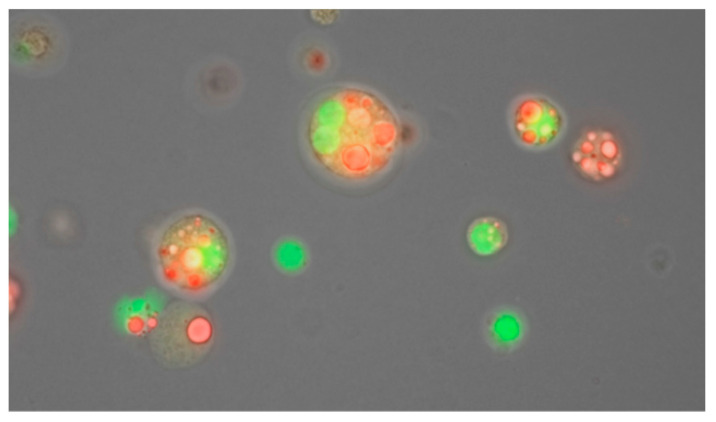
Membrane enclosed structures isolated from human milk stained with neutral lipid stain Nile red (**red**) and DNA stain Draq5 (**green**). Scale bar represents 20 μm. Image provided by Ms. Isabel Schultz-Pernice.

Cells can be isolated through centrifugation of milk and have been found to be enriched in micro-RNA (miRNA), which are regulatory biomolecules, that potentially play a role in infant development [300]. Whilst cell content of milk is variable, it has been found that the greatest number of cells can be isolated when milk samples are collected 30 min post-feed [301]. It should be noted, however, that not all milk-derived membrane enclosed structures (resembling cells) bear a nucleus, and that some are instead enlarged milk fat globules [302] (Figure 15). Why cells enter milk is still a mystery, however it has been found that a subset of cells appear to survive the infant gastrointestinal tract which may potentially impact the receiving offspring [303]. Indeed, we have shown that the immune cell compartment of milk changes depending on the health status of the mother and infant [262] which may lead to a downstream protection for the vulnerable child.

Together with immune cells, milk also contains maternal epithelial cells which through analysis may provide insights into the maturation and function of the lactating mammary gland. An early study found that prior to culture, milk cells predominantly expressed the luminal cell marker keratin 18 (CK18), and after culture the cells expressed markers associated with progenitors (CK5, Nestin) and myoepithelial cells (CK14) [304]. This culture-inducible epithelial lineage plasticity of milk cells is thought to be regulated, in part, by Sigma (14-3-3σ) [305]. Further studies by our group have found that under different culture conditions, milk cells expressed markers associated with tissues derived from all three embryonic layers [306]. Moreover, milk cells were found to express the pluripotent stem cell transcription factors OCT4, SOX2 and NANOG on a protein and mRNA level. These findings paralleled studies in non-pregnant, non-lactating breast tissue published around the same time [307,308]. Whilst the concept of pluripotent mammary stem cells existing in HM has been extensively reviewed [309] few studies have provided supporting evidence, suggesting the need for more targeted research.

In this connection, there have recently been great advances in the tools available to characterise mammary cell differentiation capabilities (i.e., stemness), such as in vivo lineage tracing studies (that allow tracking the progeny of a single cell through organ development) and technologies that allow for unbiased cell subtype identification such as single-cell RNA-sequencing/Assay for Transposase-Accessible Chromatin (scRNA-seq/scATAC-seq) [310]. Findings from many of these studies suggest that cells in the adult mammary gland are unipotent under homeostatic conditions and only acquire stem cell characteristics in artificial reprogramming environments [311] (such as ex vivo cell culture).

Increasing numbers of sc-RNA-seq studies have attempted to profile human mammary cells from different stages of development in an attempt to further unravel the subpopulations of cells that exist in the breast [312]. In line with findings from lineage tracing studies, a study examining breast tissue taken from adult non-pregnant, non-lactating individuals did not find a quiescent niche of mammary stem cells [313]. Recently, two studies have examined cells isolated from HM via scRNA-seq and found that milk contains predominantly luminal and immune cells [314,315]. Whilst both studies concur that luminal milk cells have a similar gene expression profile to luminal progenitor cells from the breast, pluripotent transcription factors were not found to be co-expressed at a detectable levels in single cells, nor expressed at higher levels than the cells isolated from the normal resting breast ([315]). Interestingly however, the milk luminal cells appear to be heterogeneous, consisting of two major luminal subtypes LC1 and LC2. In particular, one of these milk luminal cell subpopulations expressed higher levels of immunomodulatory and antigen presenting genes suggesting potential cross talk between the epithelial compartment with the microenvironment of the lactating mammary gland. Whilst many questions remain of the role and function of cells in milk, further investigation of these cells is warranted and may provide further excellent insights into the biology of milk secretion and the immune and developmental role milk plays for the breastfed infant.

## 30. Breastfeeding during COVID

As the COVID-19 pandemic continues to impact society, women have been disproportionally affected by the consequences of the virus. Mothers have faced an increasing burden of tasks around the home, parenting, educational, and broader family support roles, and make-up the most significant portion of the frontline health workforce [316]. Inconsistent and detrimental policy changes have negatively impacted pregnant and lactating women in particular. Due to uncertainty about mother-to-infant transmission of acute respiratory syndrome coronavirus 2 (SARS-Cov-2), many hospitals and governments rapidly brought in policies to prevent transmission early in the pandemic. These included restricting women’s access to support people during labour, isolating mothers with confirmed or suspected infection from their infants and discharging women early from the hospital. Moreover, families have received conflicting advice on whether to breastfeed directly or not and once at home, breastfeeding women experienced reduced access to lactation and mental health support [317,318,319].

The overwhelming evidence contradicts fears that the mother may transmit the virus to her infant via her milk [101,317] with no study to date demonstrating that SARS-Cov-2 is transmitted via HM [320,321,322]. While viral RNA has been isolated in some HM samples of positive SARS-Cov-2 mothers, no samples contained replicant-capable SARS-Cov-2, and thus infectious virus has not been found in milk [101,320,322,323,324,325]. Instead, there is evidence that antibodies to SARS-Cov-2 isolated in HM have an immunological response against the virus [326,327,328], with samples from infected mothers demonstrating strong sIgA activity specific to SARS-CoV-2. When active SARS-CoV-2 has been added to HM in the laboratory setting, holder pasteurisation (62.5 °C for 30 min) inactivates replicant-capable SARS-CoV-2. As such PDHM, when pasteurized using the Holder technique, can be considered as safe and feasible for use

Finally, with the global roll-out of vaccines against SARS-CoV-2, the accumulating evidence has demonstrated that both pregnant and breastfeeding women secrete SARS-CoV-2 specific IgA and IgG antibodies after vaccination [328,329,330] Furthermore, they had similar responses to nonpregnant controls, with immune transfer occurring via the placenta and HM. The current evidence indicates that pregnant and breastfeeding women should continue to be routinely offered vaccination against SARS-CoV-2.

Overwhelming evidence supports breastfeeding throughout the COVID-19 pandemic; however, the support enabling women to continue breastfeeding and the mental wellbeing of pregnant and lactating women remains a significant concern. Our research in Australia and New Zealand has demonstrated that during the COVID-19 pandemic, mental health issues faced by breastfeeding women appear to be exacerbated by COVID-19. We found greater health consequences noted for women who were pregnant for a longer duration during the pandemic and living in regions with higher COVID-19 infection rates [331]. International research supports this notion, with unprecedented increased postpartum anxiety and depression rates occurring during the pandemic amongst pregnant and lactating women [332,333,334]. While some breastfeeding mothers have noted the pandemic and lockdowns have resulted in less pressure and more family support at home to continue breastfeeding [331,332,335,336,337], others have highlighted a reduction in access to support has directly contributed to their early weaning [332]. More robust policies and actions that enable mothers to access their immediate support networks and mental health services remain essential to protect women’s wellbeing and continued breastfeeding as the pandemic continues.

## 31. Summary

In summary the understanding of breastfeeding and human lactation as a biological system will provide a strong knowledge base from which to direct future research, devise interventions to improve maternal and infant health as well as inform public policy with respect to breastfeeding. New research methods will create the much-needed evidence to underpin education and advocacy in this field. Indeed, human lactation science is critical to the success of the global impetus to increase breastfeeding universally.

## Figures and Tables

**Table 1:** Breastfeeding characteristics measured for 71 exclusively breastfeeding dyads in Western Australia, term infants 1 to 6 months old. Single breastfeed: breastfeed from one breast, breastfeeding session: breastfeed from more than 1 breast.

	Mean (SD)	Range
24 h milk intake	788 (169)	478–1356
Single breastfeed		
Frequency (feeds/24 h)	11 (3)	6–18
Volume transferred (mL)	76 (13)	0–240
Breastfeeding session		
Frequency (feeds/24 h)	8 (2)	4–13
Total volume transferred (mL)	101 (16)	0–350
Fat content		
Mean fat content (g/L)	41.1 (7.8)	22.3–61.6
Total fat consumed (g)	32.0 (7.7)	5.4–49.5

**Table 2:** Risk factors for low milk production.

Breast Anatomy/Genetics	Breast Physiology	Milk Removal (Autocrine Control)	
		Infant	Pump
No breast growth in pregnancy	Separation from the infant	Infrequent and ineffective breast emptying	Infrequent and ineffective breast emptying
Breast hypoplasia	Pregnancy complications	Oral anomalies-ankyloglossia	Slow stimulation of ME
Breast surgery	Maternal endocrine disorders	Prematurity	Incorrect pump settings (vacuum, vacuum pattern)
Nipple piercing	Mastitis	Infants exerting strong vacuums and causing pain	Cold conditions-reduce efficacy of milk removal
Zinc transporter mutations	Blocked ducts (temporary)	Low tone, Down’s syndrome	Pump shield not fitted correctly to maternal anatomy
	Medications	Cleft lip and/or palate	

**Table 3:** Percentages of correct estimates of milk transfer as assessed by test weighing [48]. PMA: post menstrual age.

	Infants ≥ 34/40 PMA *n* = 902	Infants < 34/40 PMA *n* = 284
Minimal or no milk transfer, give full supplement	98%	99%
Partial feed transferred, give 50% supplement	29%	16%
Full feed transferred, no supplement needed	47%	91%

**Table 4:** Factors influencing breastfeeding sucking characteristics and milk removal.

	Sucking	Milk Removal
Nipple pain	Strong intra-oral vacuums (baseline and peak) Compressive tongue motion	May or may not be impacted Most women can attain a full milk production with support
Nipple shield	Sucking characteristics do not differ significantly	Duration of feeds were longer reducing efficiency however this is not clinically significant Women using shields for persistent pain in the first week of lactation experienced no impact on milk transfer and effectiveness of breast emptying Most women can attain a full milk production with support
Ankyloglossia	Anterior tongue tie associated with tongue motion compressing either the nipple base or tip	Frenotomy for anterior tongue tie ‘normalised’ tongue movement and reduced pain improved milk production Frenotomy for posterior tongue tie reduced pain but did not improve milk production
Prematurity	Weak intra-oral vacuums (both baseline and peak) Tongue action during breastfeeding similar to term infants Tongue action with nipple shields similar to breastfeeding	Low milk volumes removed from the breast with or without a nipple shield
